# Blood TfR+ exosomes separated by a pH-responsive method deliver chemotherapeutics for tumor therapy

**DOI:** 10.7150/thno.37220

**Published:** 2019-10-14

**Authors:** Lijun Yang, Donglin Han, Qi Zhan, Xueping Li, Peipei Shan, Yunjie Hu, Han Ding, Yu Wang, Lei Zhang, Yuan Zhang, Sheng Xue, Jin Zhao, Xin Hou, Yin Wang, Peifeng Li, Xubo Yuan, Hongzhao Qi

**Affiliations:** 1 Institute for Translational Medicine, Qingdao University, Qingdao 266021, China.; 2 College of Materials Science and Engineering, Qingdao University of Science and Technology, Qingdao 266042, China.; 3 Tianjin Key Laboratory of Composite and Functional Materials, School of Materials Science and Engineering, Tianjin University, Tianjin 300072, China.; 4 School of Clinical Medicine, Weifang Medical University, Weifang 261042, China.

**Keywords:** blood TfR+ exosomes, superparamagnetic nanoparticles, pH-responsive, drug delivery, tumor therapy

## Abstract

Blood transferrin receptor-positive (TfR+) exosomes are a kind of optimized drug delivery vector compared with other kinds of exosomes due to their easy access and high bio-safety. Their application facilitates the translation from bench to bedside of exosome-based delivery vehicles.

**Methods:** In this study, a pH-responsive superparamagnetic nanoparticles cluster (denoted as SMNC)-based method was developed for the precise and mild separation of blood TfR+ exosomes. Briefly, multiple superparamagnetic nanoparticles (SPMNs) labeled with transferrins (Tfs) could precisely bind to blood TfR+ exosomes to form an exosome-based cluster due to the specific recognition of TfR by Tf. They could realize the precise magnetic separation of blood TfR+ exosomes. More importantly, the pH-responsive dissociation characteristic of Tf and TfR led to the mild collapse of clusters to obtain pure blood TfR+ exosomes.

**Results:** Blood TfR+ exosomes with high purity and in their original state were successfully obtained through the pH-responsive SMNC-based method. These can load Doxorubicin (DOX) with a loading capacity of ~10% and dramatically increase the tumor accumulation of DOX in tumor-bearing mice because of their innate passive-targeting ability. In addition, blood TfR+ exosomes changed the biodistribution of DOX leading to the reduction of side effects. Compared with free DOX, DOX-loaded blood TfR+ exosomes showed much better tumor inhibition effects on tumor-bearing mice.

**Conclusion:** Taking advantage of the pH-responsive binding and disaggregation characteristics of Tf and TfR, the SMNC-based method can precisely separate blood TfR+ exosomes with high purity and in their original state. The resulting blood TfR+ exosomes showed excellent bio-safety and enable the efficient delivery of chemotherapeutics to tumors, facilitating the clinical translation of exosome-based drug delivery systems.

## Introduction

Exosomes have attracted increasing attention in recent years due to their huge potential in the field of drug delivery 1,2. Since the first report on the delivery of exogenous siRNA 3, various therapeutic drugs, such as chemotherapeutics 4,5, sonosensitizers 6, small-molecule inhibitors 7, proteins 8,9, and genes 10, have been delivered by exosome-based delivery vehicles. The state-of-art CRISPR/Cas9 system has also been successfully transported through exosomes recently 11,12. Although great progress has been made, the clinical translation of these systems has still been a big challenge. The number of exosomes separated from current sources, such as cell culture medium, is extremely limited, hindering their mass production 13. In addition, the bio-safety of these exosomes is modest since they often contain oncogenic or immunogenic components 14,15. Therefore, it is necessary to choose the appropriate kind of exosomes as optimized vectors to facilitate their translation from bench to bedside.

The blood of healthy animals contains plenty of transferrin receptor-positive (TfR+) exosomes, which are without immune-stimulating activity and cancer-stimulating properties 16. For example, reticulocytes (RTCs) release ~10^14^ (at least 200 μg) TfR+ exosomes during their maturation into erythrocytes 17. Their easy access and high bio-safety imply that blood TfR+ exosomes can be a promising alternative delivery vector. In addition, the successful blood transfusion between people of the same blood types further proves the operability and clinical translation potential of allogeneic blood TfR+ exosomes for drug delivery 16. However, there are no reports on blood TfR+ exosomes-based drug delivery until now because the classical methods for exosomes separation cannot realize the precise separation of blood TfR+ exosomes. Exosomes separated by the “gold standard” ultracentrifugation method are frequently contaminated with other proteins and particulates 18. Combining differential ultracentrifugation with a density-based technique helps remove these contaminants, but the non-ideal clumping of the exosomes is unavoidable 19. Commercial precipitants, such as ExoQuick Precipitation Solution, can be adversely affected by contaminating proteins, which require additional filtration or ultracentrifugation steps for removal 20. High-performance liquid chromatography on a gel exclusion column (HPLC-GEC) is also non-specific because of the size dependence of column packing materials. In addition, the interaction of exosomes with the elution buffer can cause them to degrade or aggregate, resulting in poor fractionation and decreased yields 19. Taking advantage of the physicochemical characteristics of exosomes, such as viscoelasticity, many novel methods can obtain exosomes without the contamination of proteins or other kinds of vesicles 21. However, further improvements in the ability to separate specific kinds of exosomes are needed. Therefore, exploiting an advanced method for the precise separation of blood TfR+ exosomes is the precondition for drug delivery.

Magnetic particle-based or microfluidic-based magnetic separation methods, which depend on the surface composition of exosomes, have the potential for the precise separation of blood TfR+ exosomes. These methods have been utilized to separate specific kinds of exosomes, such as epithelial cell adhesion molecule (EpCAM) positive exosomes 22 and epidermal growth factor receptor (EGFR) positive exosomes 23. However, the mild release of these exosomes from magnetic particles or microfluidic tubes has never been considered seriously. The commonly used methods for the dissociation of antibody and antigen, such as very low pH conditions (<3), would damage the structure and function of exosomes 24,25. In addition, the large size of magnetic beads may also result in the destruction of exosomes. For example, Clayton et al. have proved that two or more exosomes potentially fuse together at the magnetic bead surface 26. Hence, developing an upgraded magnetic separation method is an appropriate way to separate blood TfR+ exosomes for drug delivery.

**Scheme 1:**
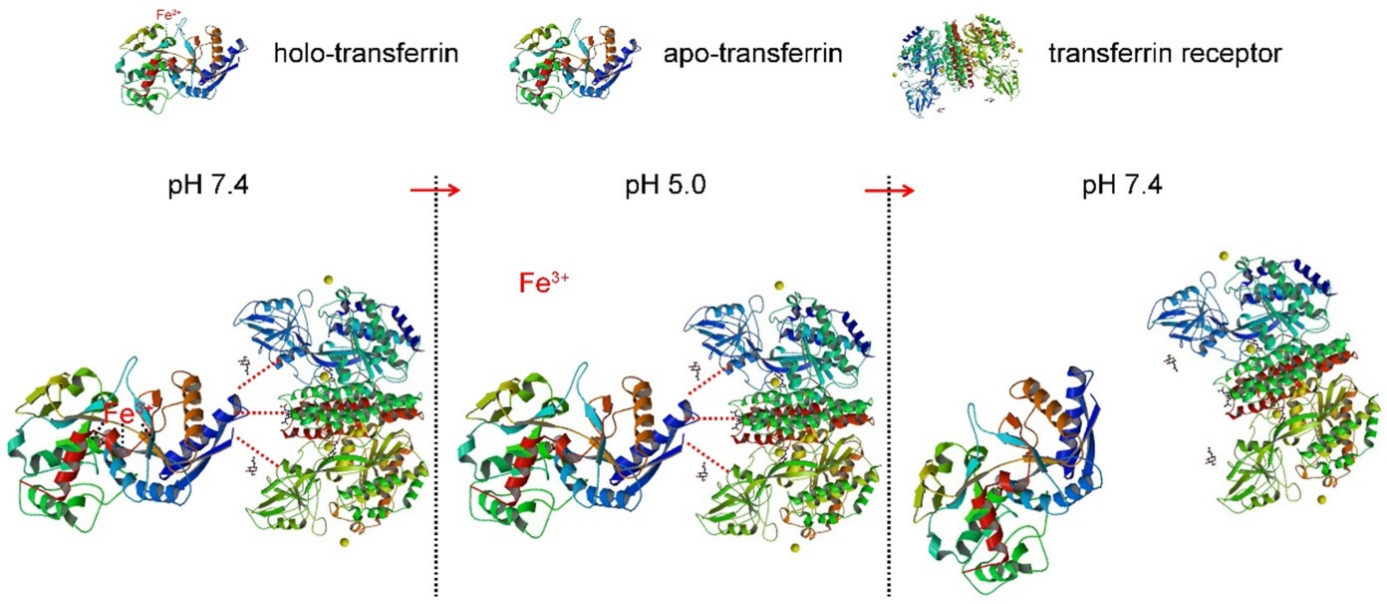
The dissociation characteristic of transferrin and transferrin receptor.

Here a superparamagnetic nanoparticles cluster (denoted as SMNC)-based method is developed for the precise and mild separation of blood TfR+ exosomes. In this upgraded immunomagnetic separation method, superparamagnetic nanoparticles (SPMNs) are adopted to reduce the potential risk to exosomes due to their small size. In addition, transferrin (Tf) is used as the ligand to obtain blood TfR+ exosomes. The specific recognition of TfR by Tf can realize the precise magnetic separation of blood TfR+ exosomes. More importantly, the dissociation of Tf and TfR is responsive to pH 27. As shown in Scheme 1, holo-transferrin (contain ferric ion) could combine with TfR at pH 7.4. When pH is changed to 5.0, holo-transferrin releases ferric ion to form apo-transferrin (ferric ion-free). However, apo-transferrin still combines with TfR until the pH is changed to 7.4 again. In view of the pH of the medium surrounding exosomes being shifted from 5.0 (in the multi-vesicular body) to 7.4 (in the extracellular space) during their formation process 28, we deduce the pH change between 5.0 and 7.4 would not damage exosomes. Therefore, the pH-responsive binding and dissociation characteristics of Tf and TfR can be used for the mild release of exosomes from SPMNs.

**Scheme 2:**
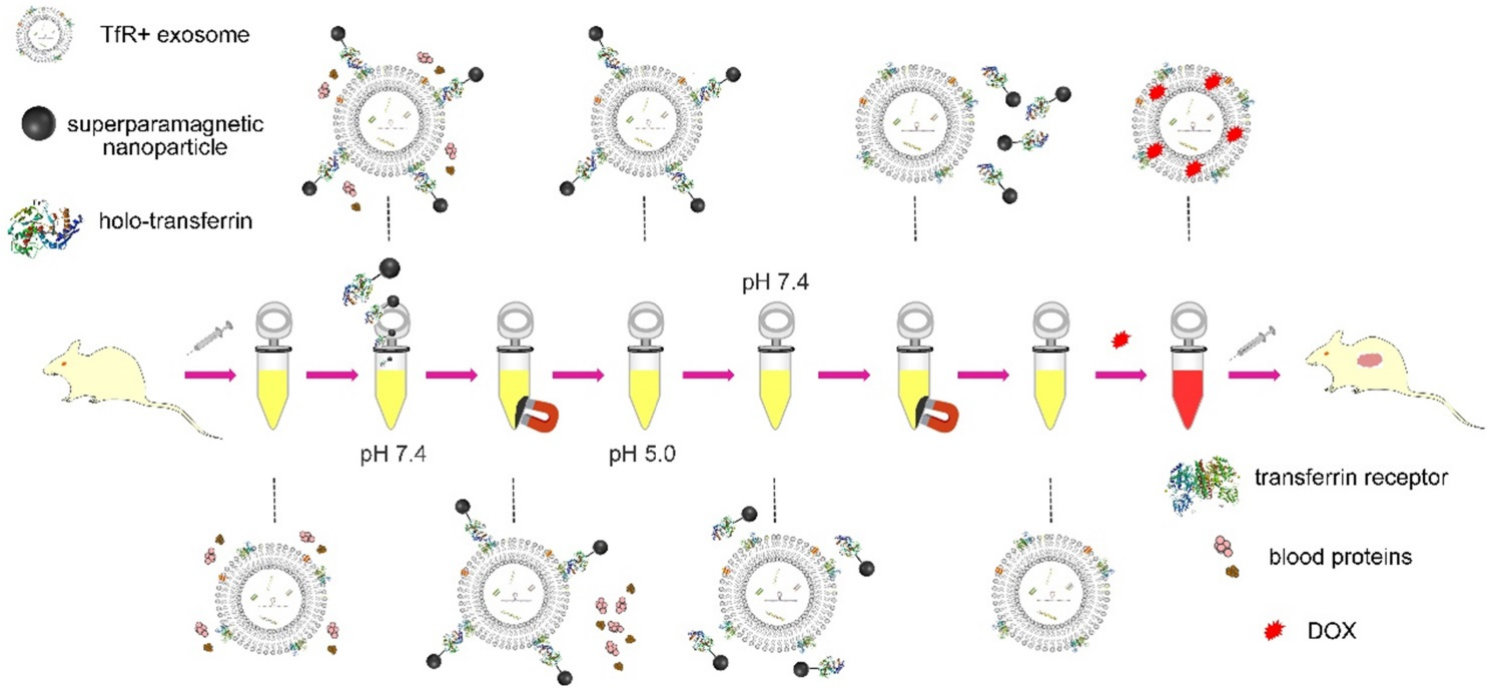
Schematic illustration of the separation of blood TfR+ exosomes for tumor-targeting drug delivery by the pH-responsive method.

As shown in Scheme 2, holo-transferrin is chosen as the ligand and is labeled on SPMNs. SPMNs can combine with blood TfR+ exosomes through the interaction of Tf and TfR to form SPMN-exosome complexes (denoted as SMNC-EXOs) at pH 7.4. Under an external magnetic field, SMNC-EXOs can be separated, and they are easily re-dispersed once the magnetic field is removed. Although holo-transferrin transforms to apo-transferrin when the pH is changed to 5.0, SMNC-EXOs are still stable. Only when the pH is changed to 7.4 again, are they dissociated, and pure blood TfR+ exosomes (denoted as M-EXOs) can be obtained through magnetic removal of free SPMNs. The drug delivery potential of the resulting M-EXOs is confirmed using doxorubicin (DOX) as the model drug. The DOX-loaded M-EXOs (denoted as D-M-EXOs) show excellent *in vivo* tumor suppression. The SMNC-based method for the first time realizes the precise and mild separation of blood TfR+ exosomes, facilitating the clinical translation of exosome-based drug delivery. Furthermore, this methodology can inspire other researchers to exploit new ways for the exosomes separation from animal body fluid.

## Experimental section

### Materials and reagents

Carboxyl-group functionalized superparamagnetic Fe_3_O_4_ nanoparticles were purchased from Nanjing Nanoeast Biotech Co., Ltd. Holo-transferrin, Fluorescein isothiocyanate (FITC), Doxorubicin hydrochloride (DOX), triethylamine, 1-ethyl-3-(3-dimethylaminopropyl) carbodiimide hydrochloride (EDC), sulfo-NHS and 2-mercaptoethanol were purchased from Sigma-Aldrich. A Bicinchonininc acid (BCA) protein assay kit and an enzyme-linked immune-sorbent (ELISA) assay kit were both purchased from Thermo Scientific. A Cell Counting Kit-8 was purchased from Dojindo Molecular Technologies Inc. NHS-Cy 5.5 was purchased from ApexBio Technology.

### The modification of SPMNs with Tf

Carboxyl-group-functionalized superparamagnetic Fe_3_O_4_ nanoparticle solution (40 μL, 2.5 mg/mL) was mixed with EDC and sulfo-NHS at a molar ratio of 1:2:3 (pH 5.5), and the mixture was incubated at room temperature for 1 h. Then, 1 μL of 2-mercaptoethanol was added to terminate the reaction. The activated superparamagnetic Fe_3_O_4_ nanoparticles were magnetically separated and were re-suspended in 200 μL of borate buffer (20 mM, pH 8.5). Then, 10 μg of holo-transferrin was added, and the mixture was incubated for 12 h at 4°C under nitrogen. Finally, the SPMN-Tf complexes were purified by magnetic separation and washed three times with PBS. The resulting solution (200 μL) was stored at 4°C.

### The separation of SMNC-EXOs from serum

First, 1 mL of serum was added to an ultrafiltration tube (Millipore, 100kDa) and centrifuged at 4000×g for 30 min at 4°C. Then, the serum solution was mixed with SPMN-Tfs solution and blended homogeneously using a vortex shaker. This mixture was incubated for 1 h at 37°C. The products were obtained after 40 min of magnetic separation and were washed three times with PBS. The resulting SMNC-EXOs were re-dispersed in PBS and were stored at 4°C until they were used.

### The separation of M-EXOs

To obtain TfR+ exosomes, the stored SMNC-EXOs solution (pH 7.4) was firstly dialyzed against acetate buffer (pH 5.0) for 12 h at 4°C. The dialyzate was changed every 2 h. Then the dialysate was changed to PBS (pH 7.4) again, and the SMNC-EXOs solution was dialyzed for 12 h at 4°C. The dialyzate was also changed every 2 h. After dialysis, free SPMNs were magnetically separated (magnetic field intensity: 1T) and the supernatant was collected. M-EXOs dispersed in the supernatant were stored at 4°C until they were used.

To determine the separation efficiency of exosomes, the change in CD63 concentration during the separation process was tested. The concentrations of CD63 in SMNC-EXOs solution (pH 7.4 and pH 5.0, separated from 1 mL of serum) and in the separated M-EXOs (pH 7.4, separated from 1 mL of serum) were measured by using a CD63 ELISA kit. 1 mL of PBS and 1 mL of serum were used as negative control and positive control.

### The measurement of the ferric ions concentration

First, 300 μL of fresh separated SMNC-Tf solution and SMNC-EXOs solution were both divided into three equal parts (100 μL) and were placed into solution with different pH values (7.4, 5.0, and 7.4). After 12 h, they were mixed with 100 μL of 10 mM HCl and 100 μL of the iron-releasing reagent (1.4 M HCl and 4.5%(w/v) KMnO_4_ in H_2_O). These mixtures were incubated for 2 h at 60°C. After the mixtures had cooled to room temperature, 30 μL of the iron-detection reagent (6.5 mM ferrozine, 6.5 mM neocuproine, 2.5 M ammonium acetate, and 1 M ascorbic acid dissolved in H_2_O) were added to each solution. After 30 min, each solution was transferred into a well of a 96-well plate, and the absorbance was measured at 550 nm on a microplate reader. The iron content of the sample was calculated by comparing its absorbance to that of a range of standard concentrations of equal volume that had been prepared in a way similar to that of the sample (mixture of 100 μL of FeCl_3_ standards (0-128 μM) in 10 mM HCl, 100 μL 50 mM NaOH, 100 μL releasing reagent, and 30 μL detection reagent).

### The characterization of M-EXOS

The size and number of M-EXOs were determined by recording and analyzing the Brownian motion of particles using a NanoSight NS300 system and Nanoparticles Tracking Analysis (NTA) software (Malvern, Worcestershire, United Kingdom). Zeta potentials of the M-EXOs were measured by dynamic light scattering (BI-90Plus, Brookhaven Instruments Ltd., USA), and their morphology was visualized using a high-resolution transmission electron microscope (TEM, JEM-2100F, JEOL Ltd., Japan) and scanning electron microscope (SEM, FEI Quanta 200, USA).

The obtainment of M-EXOs was confirmed by western blot analysis. Briefly, M-EXOs were lysed, and lysates were separated and transferred to PVDF membranes. Membranes were rinsed with PBS for several minutes and blocked with Odyssey blocking buffer for 1 h at 22°C. Then, they were incubated with primary antibodies against CD9, CD63, CD81, and TfR (Zhongshan Bio Corp, Beijing, China), followed by incubation with fluorescent secondary antibodies (Zhongshan Bio Corp, Beijing, China). Images were acquired with an Odyssey infrared imaging system and analyzed using software specified by the Odyssey systems. As a control group, exosomes separated by ultracentrifugation (denoted as UC-EXOs) were manipulated according to the abovementioned method.

### The cellular uptake of M-EXOs

Confocal fluorescence microscopy was used to assess the intracellular trafficking of M-EXOs. Cells that had grown on the glass coverslips (pretreated with polylysine) of a six-well plate were incubated with FITC-labeled M-EXOs (FITC-M-EXOs) for 24 h. Following incubation, the cells were washed three times with PBS and fixed in paraformaldehyde for 15 min. Localization of FITC-M-EXOs in cells was visualized using a confocal microscope (Carl Zeiss Microscope Systems, Jena, Germany) with identical settings for each confocal study. To quantify the cellular uptake efficiency, FITC signal uptake rates were detected using flow cytometry (Becton, Dickinson and Company, USA).

To assess the intracellular trafficking of M-EXOs, H22 cells grown on the glass coverslips of a 6-well plate were incubated with FITC-M-EXOs for 4 h. Then, the cells were incubated with culture medium containing 50 nM of LysoTracker blue DND-22 for 0.5 h. The cells were then washed three times with PBS and localization of FITC-M-EXOs in cells was visualized by confocal microscopy with identical settings for each confocal study. In addition, FITC signal uptake rates were detected using flow cytometry (Becton, Dickinson and Company, USA).

### Cell viability assay

The cytotoxicity of M-EXOs in H22 cells was evaluated using a Cell Counting Kit-8. First, 4000 cells were seeded into 96-well plates and grown in complete medium at 37°C for 24 h. Subsequently, the culture medium in each well was replaced with a fresh medium that contained M-EXOs in a series of concentrations. Cells without the addition of M-EXOs were used as a control group. Each group included six replicates. After culturing for an additional 48 h, CCK solution was added and cell viability was calculated as the ratio of the absorbance of test and control wells. The absorbance was measured at 450 nm using a microplate reader.

### Blood compatibility assay

First, 4 mL of mice whole blood was added to 8 mL of saline, and red blood cells (RBCs) were isolated by centrifugation at 1,000 g for 15 min. RBCs were washed five times with sterile saline solution. Following the final wash, the RBCs were diluted with 40 mL of saline. Then, 0.2 mL of the diluted RBC suspension was added to 0.8 mL of M-EXOs to achieve final M-EXOs concentrations of 10, 100, and 1,000 μg/mL. The suspension was vortexed briefly before leaving it under static conditions at room temperature for 4 h. Thereafter, the mixture was vortexed briefly again and centrifuged at 1,000 g for 10 minutes. Next, 400 μL of supernatant was measured using UV-Vis absorbance spectrum scanning. After that, 0.2 mL of diluted RBC suspension, which was incubated with 0.8 mL of saline and 0.8 mL of distilled water, was used as the negative or positive control.

### Drug loading and releasing

DOX was used as the model drug. First, 20 μL of DOX solution (2 mg/mL) was added to the M-EXOs solution (200 μL, 1 mg/mL) with moderate stirring. After 30 min, 5 μL of triethylamine was added, and then the solution was stirred 1 h. D-M-EXOs were obtained *via* magnetic separation at 4°C. The amount of DOX that was loaded into the M-EXOs was calculated from a calibration curve acquired from UV-Vis spectrophotometer measurements based on the absorbance intensity at 485 nm. The release of DOX was performed as described previously with slight modifications. In short, 4 mL of D-M-EXOs solution was transferred into a dialysis tube (molecular weight cut-off: 14 kDa). The tube was first placed into 10 mL of PBS buffer (pH 7.4). After 10 h, the tube was placed into 10 mL of acetate buffer (pH 5.0). The release of DOX was performed at 37°C. At selected time intervals, the dialysate was removed for UV-Vis spectrophotometer analysis and replaced with a fresh buffer solution. The concentrations of DOX were determined according to standard curves at the corresponding buffer solutions.

### Inhibition of tumor cells by D-M-EXOs

The tumor inhibition effects of DOX and D-M-EXOs were evaluated using a CCK-8. First, 4000 H22 cells or 4T1 cells were seeded into 96-well plates and grown in complete medium at 37°C for 24 h. Subsequently, the culture medium was replaced with complete medium containing 0, 0.25, 0.5, 1, 2, 4, 8, 10, and 12 µg/mL of DOX. The same procedure was carried out to study the influence of D-M-EXOs (with equivalent concentrations of DOX) on cell viability. At 48 h, CCK-8 solution was added and cell viability was assessed. The cells without treatment were used as the control and cell viability was calculated as the ratio of the absorbance of the test and control cells.

Confocal fluorescence microscopy was used to assess the intracellular trafficking of M-EXOs and DOX. Cells that had grown on the glass coverslips of a six-well plate were incubated with DOX and FITC-labeled D-M-EXOs for 24 h. Following incubation, the cells were washed three times with PBS and fixed in paraformaldehyde for 15 min. Localization of FITC-M-EXOs and DOX in cells was visualized using confocal microscopy with identical settings for each confocal study.

The H22 and 4T1 cells were seeded in a dish at a density of 5×10^5^ cells/mL for 24 h to bring the cells to the desired confluence. The medium was replaced with fresh medium containing different drugs, and the cells were incubated for 48 h. Then protein lysates were separated using SDS-PAGE gel and were transferred onto PVDF membranes, then incubated with primary antibodies that could detect caspase-3 and Bcl-2 (1:1000 dilution, Zhongshan Bio Corp.), followed by incubation with a secondary antibody (1:1000 dilution, Zhongshan Bio Corp.). The density of target protein signals was visualized by a chemiluminescent imaging system (Syngene G: BOX Chemi XR5) using an enhanced chemiluminescent detection kit.

### *In vivo* bio-distribution of D-M-EXOs

To investigate the bio-distribution of D-M-EXOs and the change in bio-distribution of DOX by M-EXOs, free DOX and Cy5.5 labeled D-M-EXOs (Cy5.5-D-M-EXOs) were injected intravenously into tumor-bearing mice. D-M-EXOs were labeled by NHS-Cy5.5 (mass ratio of 1000:1) in pH 8.5 buffer solution. Kunming mice, four- to six-weeks-old, were purchased from Charles River (Beijing, China). H22 cells were suspended in serum-free DMEM medium and inoculated subcutaneously to the flank of mice (2×10^6^ cells per mice). All animal experiments were performed according to the protocols approved by the Institute Animal Care Committee. After tumors had grown to ~100 mm^3^, the mice were divided randomly into three groups. One group was injected intravenously with Cy5.5-D-M-EXOs solution (5 mg/mL, 200 μL per mice). Another group was injected intravenously with DOX solution (0.5mg/mL, 200 μL per mice). The control group was injected with PBS (200 μL). After 24 h, whole-animal imaging was recorded using an IVIS Spectrum imaging system (IVIS 100, USA). After that, the mice were euthanized by cervical dislocation. Tumors and major organs were harvested, washed with PBS, and placed in a dish. Next, fluorescence imaging results and fluorescence intensities were recorded using an IVIS Spectrum imaging system. In addition, the tumors and major organs were harvested, washed with PBS, stored in liquid nitrogen, and triturated in mortar. The powder was then dissolved in 1 mL of borate buffer solution and ultrasonically lysed. After 30 min, 1 mL of chloroform was added to the solution, and the mixed solution was shaken for 30 min. Finally, the solution was allowed to remain stationary, and the lower solution was absorbed. According to the absorption standard curve of DOX, the absorbance was measured at 480 nm to determine the DOX content.

The distribution of D-M-EXOs in the tumor tissue was also confirmed. Kunming mice bearing H22 tumor were separated into three groups with six mice in each group. A single-dose of either PBS, DOX, or FITC-D-M-EXOs (5 mg/mL, 200 μL per mice) was injected *via* the tail vein. After 24 h, tumor tissues were isolated and were embedded in optimal cutting temperature (OCT) compound. Then, they were frozen rapidly at -20°C for 24 h. Tumor tissues were cut into 8 μm histology slices using a cryostat. Each section was dyed with 4',6-diamidino-2-phenylindole (DAPI) and covered with a coverslip. The frozen sections were observed using a fluorescence microscope from Olympus Corporation (FV1200, Tokyo, Japan).

### Cardiotoxicity and hepatotoxicity of D-M-EXOs

Kunming mice, four- to six-weeks-old, were divided randomly into three groups. One group was injected intravenously with a solution of D-M-EXOs (~100 μg of DOX) and one group was injected intravenously with DOX solution (100 μg of DOX). The control group was injected with PBS. After 48 h, blood was collected through the tail cut. Then, the mice were euthanized, and livers and kidneys were harvested. The serum levels of aspartate aminotransferase (AST), alanine aminotransferase (ALT), alkaline phosphatase (ALP), creatine kinase (CK), creatine kinase-MB (CK-MB), and creatine kinase (LDH) were determined using commercially available ELISA kits. The organs were stored overnight in 2.0% (V/V) formaldehyde solution in PBS and were then washed twice with PBS to remove excess formaldehyde. Paraffin-embedded tissue sections were stained with hematoxylin and eosin (H&E) and observed through a microscope.

### *In vivo* antitumor efficiency of D-M-EXOs

H22 or 4T1 cells were suspended in serum-free DMEM medium and were inoculated subcutaneously to the flank of the mice (2×10^6^ cells per mice). After tumors had grown to ~100 mm^3^, the mice were divided randomly into three groups (PBS, DOX, and D-M-EXOs). Each group had five mice. Solutions were administered by intravenous injections every three days (5 mg of DOX/kg of body weight per dose) for three weeks. Tumor volume was measured from: volume = length×width^2^/2. The mice were euthanized and the tumors were harvested. The tumors were photographed and their average masses were measured. Immunohistochemistry (IHC) was performed for analyzing the expression levels of Caspase-3 and Bcl-2. For the observation of tumor cell apoptosis, tumor slices were stained with H&E and terminal deoxynucleotidyl transferase (TdT)-mediated dUTP nick end labeling (TUNEL), respectively.

### Statistical analysis

Statistical comparisons were achieved using a one-way ANOVA with a Dunnett post-hoc test using GraphPad Prism 6.0 software.

## Results

### pH-responsiveness of SMNC-based method

**Figure 1:**
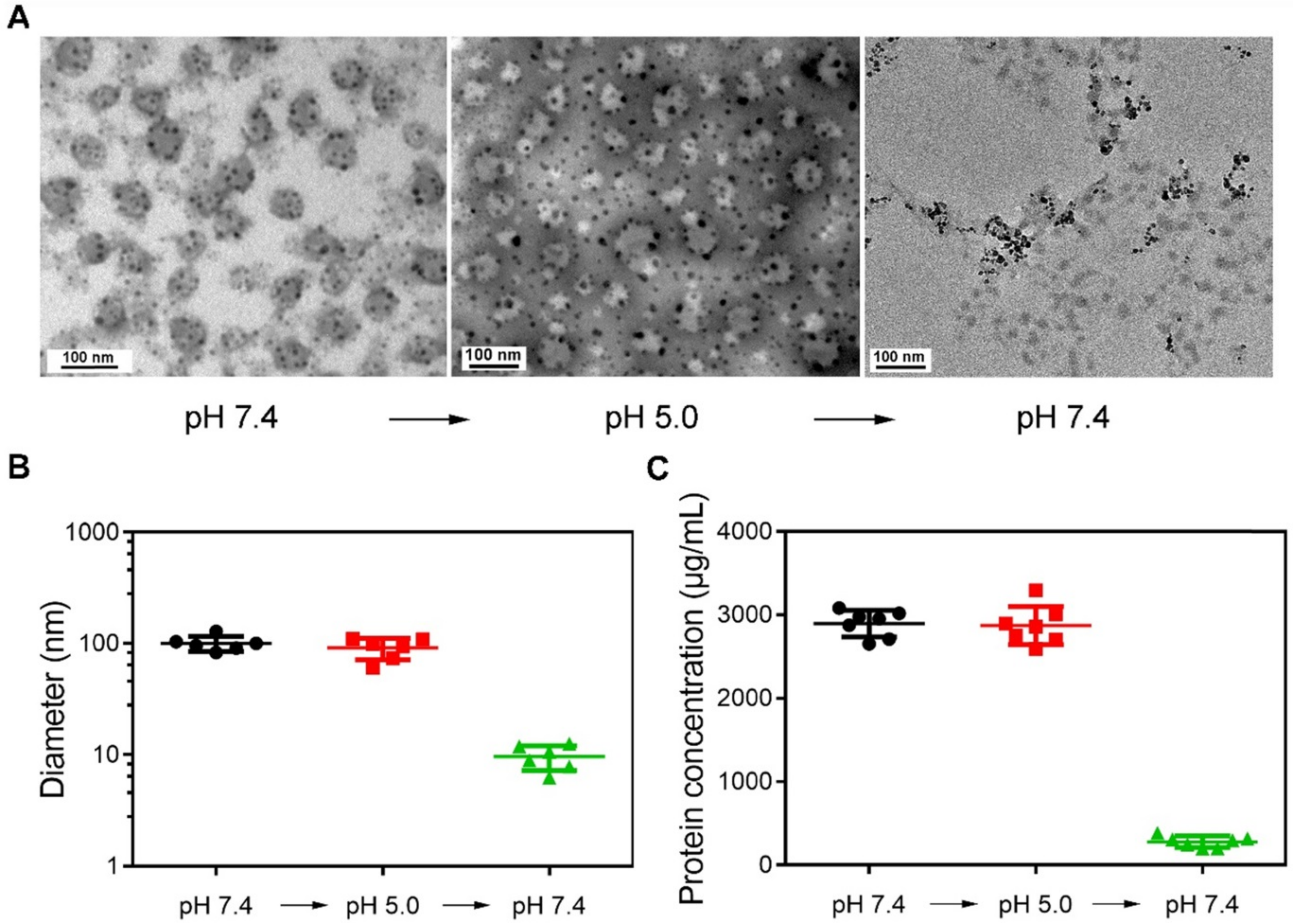
** The pH-responsiveness of SMNC-based method.** (A) Representative TEM images of magnetically separated samples with the change of pH. (B) The diameter of magnetically separated samples at different pH values. (C) The protein concentration of magnetically separated samples at different pH values.

Figure 1A shows the representative TEM images of magnetically separated samples at different pH values. The morphology of samples magnetically separated from serum (pH 7.4) was first observed. Dark spots surround spherical vesicles representing the cluster structures were formed during synthesis. When the pH was changed to 5.0, the structure of clusters was still stable. However, spherical vesicles disappeared in magnetically separated samples when pH was changed to 7.4. This may be because SPMNs were dissociated from blood TfR+ exosomes, and the re-dispersed magnetically separated products were actually SPMNs. The size of samples at different pH values was also detected (Figure 1B). The mean size of initial samples (pH 7.4) was ~100.36 nm and was similar to that of samples (~91.26 nm) re-dispersed in pH 5.0 solution. However, when samples were again re-dispersed in pH 7.4 solution, the mean size of magnetically separated samples was ~9.65 nm. These samples may be SPMNs since the mean size of commercial SPMNs was ~10 nm. In addition, the protein concentration of samples at different pH values was also measured (Figure 1C). The protein concentration of samples in pH 7.4 and pH 5.0 solutions was similar and it was dramatically reduced when samples were again re-dispersed in pH 7.4 solution. These results imply that SMNC-EXOs were formed in pH 7.4 serum and were stable even when they were dispersed in pH 5.0 solution. However, when they were again re-dispersed in pH 7.4 solution, SMNC-EXOs disintegrated, proving the pH-responsiveness of the SMNC-based method.

To further demonstrate the pH-dependent separation of TfR+ exosomes, the ferric ions concentration changes during the preparation process were tested by a colorimetric ferrozine assay. Figure S1-A was the absorbance of the Fe^2+^-ferrozine complex formed with increasing concentration of the standard FeCl_3_. The increase in absorbance was linear between 0.5-128 μM (A) and between 0.5-4 μM (B) of FeCl_3_. Furthermore, the absorbances at 550 nm of Fe^2+^-ferrozine complex in SPMN-Tfs solution and SMNC-EXOs solution were both measured. As shown in Figure S1-B, the pH change from 7.4 to 5.0 and again to 7.4 dramatically reduced the absorbance since ferric ions have been released from Tfs. According to the standard curve, the ferric ions concentration in SPMN-Tfs solution at initial pH 7.4 was ~6 μM, which was similar to the theoretical value (5.2 μM). However, the ferric ions concentration in SMNC-EXOs solution at an initial pH of 7.4 was ~40 μM, which was much higher than the theoretical value. This may be attributed to the composition of TfR+ exosomes. For example, TfR+ exosomes also contained holo-transferrins 29. In addition, it should be specially noted that the ferric ions concentration at pH 5.0 was obviously higher than that a later pH 7.4. We deduced that SPMNs would release ferric ions in acidic conditions and disturb the measurement 30. Regardless, the pH changes during the preparation process reduced the ferric ions concentration of the solution, which would induce the dissociation of TfR and Tf, indicating the separation of TfR+ exosomes was pH-dependent.

To determine the separation efficiency of exosomes, the change in CD63 concentration during the separation process was tested. As shown in Figure S2, we measured the concentration of CD63 in 1 mL serum (positive control), in SMNC-EXOs solution (pH 7.4 and pH 5.0), and in the separated M-EXOs (pH 7.4). The results show that the total concentration of CD63 in 1 mL serum is ~106.44 pg/mL and is ~52 ± 2 pg/mL in SMNC-EXOs solution (both at pH 7.4 and pH 5.0). A calculation shows that ~50% of total blood exosomes can be separated, and they are blood TfR+ exosomes. Furthermore, the total concentration of CD63 in M-EXOs solution is ~40.43 pg/mL, which demonstrates that 70%-80% of blood TfR+ exosomes can be separated from SMNC-EXOs solution by this pH-responsive method.

### The characterization of M-EXOs

**Figure 2:**
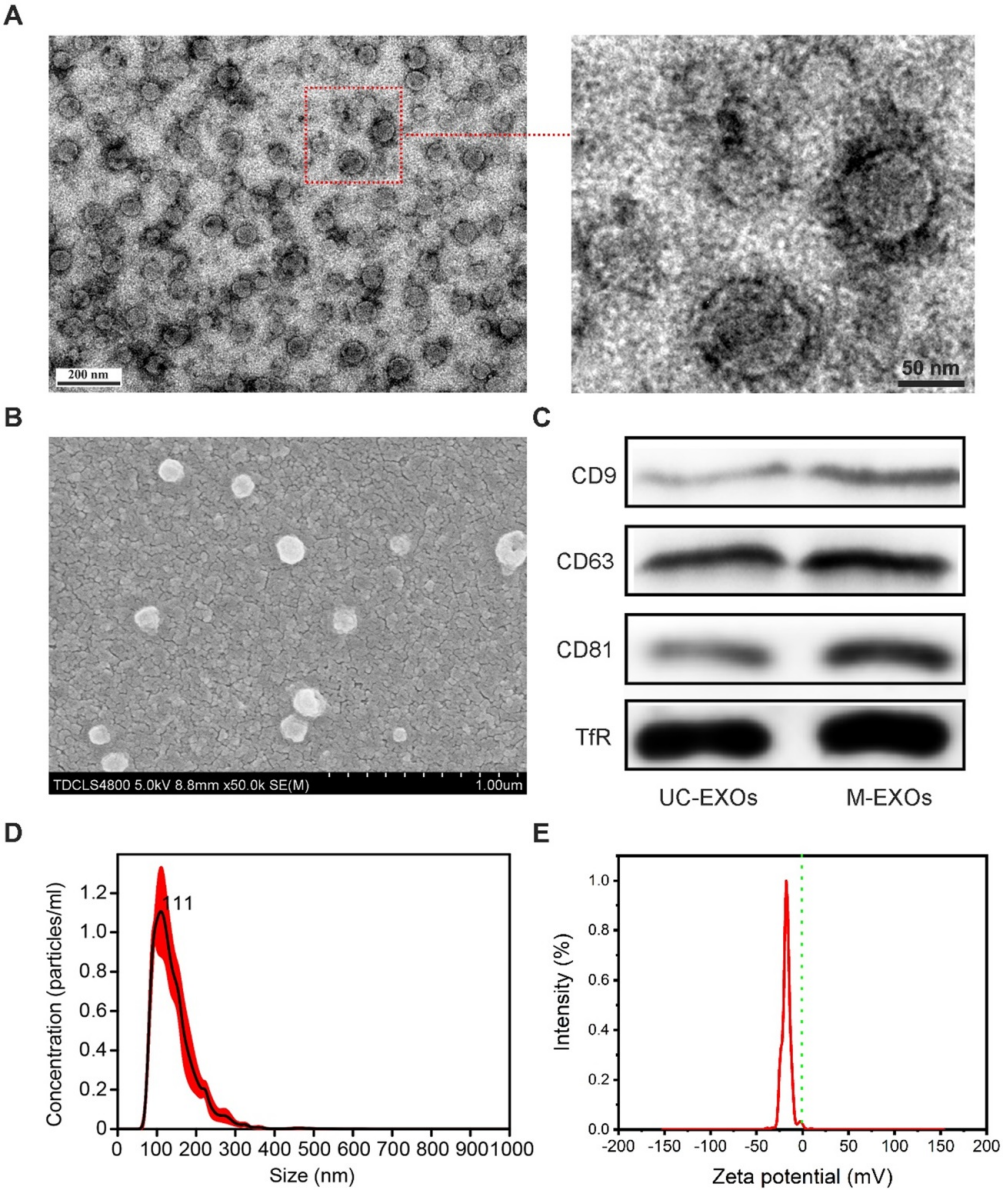
** The characterization of M-EXOs.** (A) Representative TEM images of M-EXOs. (B) Representative SEM image of M-EXOs. (C) Western blot analysis of specific exosome marker proteins (CD9, CD63, and CD81) and TfR in M-EXOs solution. (D) The size distribution of M-EXOs. E) The zeta potential of M-EXOs.

To further test whether a change in pH can lead to the disintegration of SMNC-EXOs, the morphologies of M-EXOs were characterized. As shown in the representative TEM images (Figure 2A), a typical spherical structure was observed in M-EXOs solution, implying the existence of exosomes. The representative SEM image also proved the existence of spherical vesicles (Figure 2B). The specific marker proteins of exosomes (CD9, CD63, and CD81) were detected in M-EXOs solution (Figure 2C), and exosomes separated by ultracentrifugation (denoted as UC-EXOs) were used as a control, proving the spherical vesicles were exosomes. The existence of TfR proved that these exosomes were TfR+ exosomes. Nanoparticles Tracking Analysis (NTA) was performed to further characterize the separated exosomes. As shown in Figure 2D, the size distribution of M-EXOs was centered at approximately 111 nm. Furthermore, the concentration of M-EXOs was estimated to be 1.05×10^12^ ± 1.59×10^11^ particles/mL serum. In addition, the mean zeta potential of M-EXOs was ~-17 mV (Figure 2E). The negative zeta potential was beneficial to the drug delivery of TfR+ exosomes since nanoparticles with too high or too low surface potential were easily cleared by the immune system 31.

To prove the high purity of M-EXOs, we compared M-EXOs with exosomes separated by ultracentrifugation and commercial precipitant. Figure S3-A shows the representative TEM images of exosomes separated by ultracentrifugation and commercial precipitant. Exosomes separated by ultracentrifugation were contaminated with proteins (indicated by the red arrows). Besides proteins, exosomes separated by commercial precipitant were also contaminated with large-size vesicles (indicated by the red arrows). These results imply that M-EXOs had a higher purity. To further prove this conclusion, we compared the amount of CD63 with that of total proteins in the exosomes solution (both separated from 1 mL of serum) (Figure S3-B). The ratio of the CD63 amount to the total proteins amount was the highest in M-EXOs solution, indicating their high purity. In addition, we investigated the stability of exosomes separated by different methods. At 4℃, the mean size of exosomes increased with the increase of storage time (Figure S3-C). This may be because of the aggregation of proteins or exosomes. Comparatively speaking, the size increase amplitude of M-EXOs was the smallest, further implying their high purity and high stability.

### The cellular uptake and bio-safety of M-EXOs

**Figure 3:**
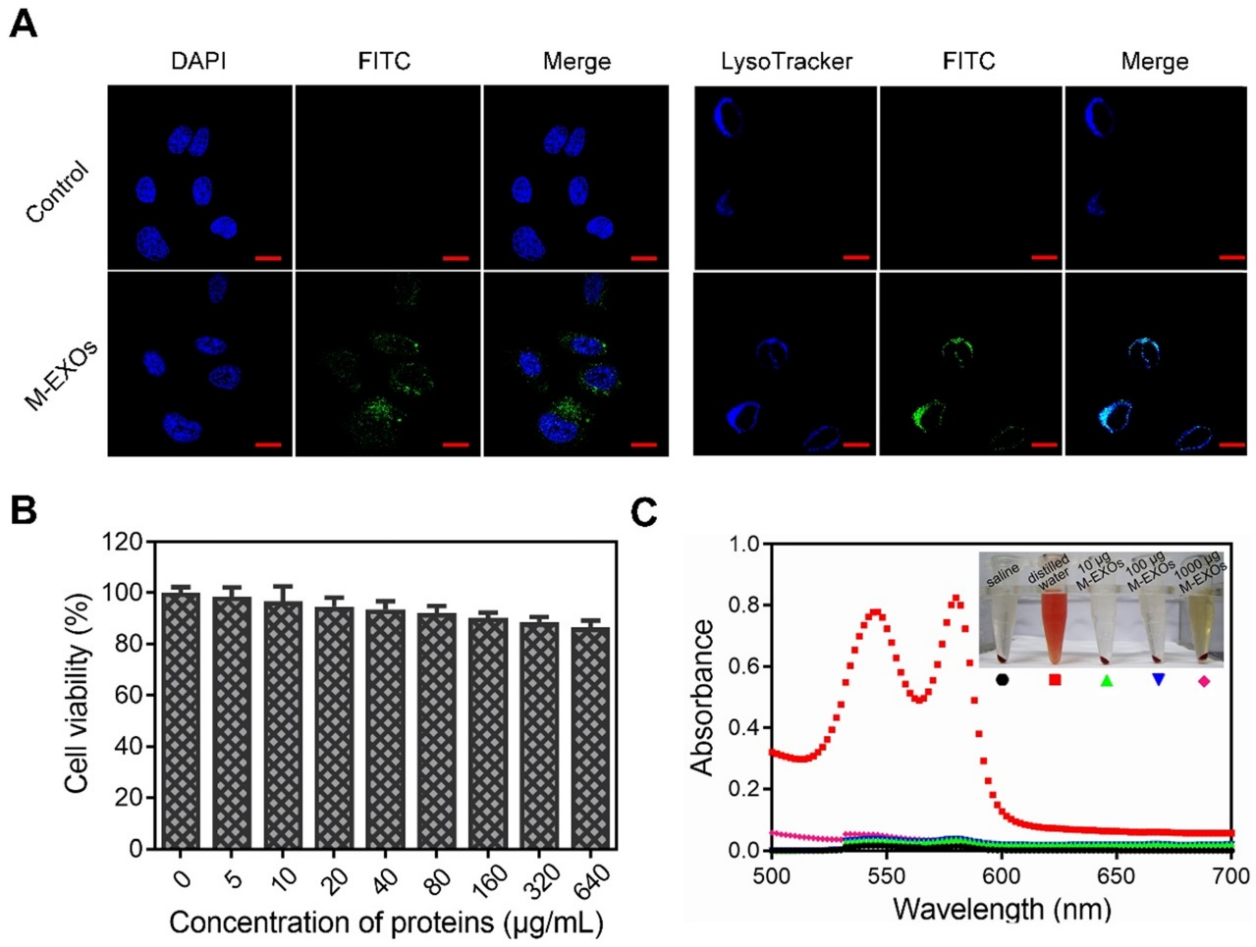
** The cellular uptake and bio-safety of M-EXOs.** (A) Intracellular distribution in H22 cells of M-EXOs (exosomes were labeled with FITC). The bar is 200 μm. (B) Cytotoxicities of different concentrations of M-EXOs as examined in H22 cells by CCK-8 assay. (C) Hemolytic activities of M-EXOs and comparison with distilled water (positive control) and saline (negative control).

The cellular uptake of the M-EXOs was tested. Protein concentration was used to denote the content level of exosomes, similar to other researchers 3. After 4 h incubation, the fluorescence signal of M-EXOs was detected in the cytoplasm (Figure 3A, left picture). Quantitative results showed that the cellular uptake efficiency of M-EXOs was respectively 35.20% and 75.13% at 100 μg/mL and 200 μg/mL (Figure S4). These results demonstrated the cellular uptake of blood TfR+ exosomes was concentration-dependent. The intracellular distribution of M-EXOs was also observed. We firstly labeled endosomes/lysosomes with LysoTracker. The fluorescence signal of M-EXOs was matched to that of endosomes/lysosomes (Figure 3A, right picture). A previous study has shown that exosomes were recruited to endosomes by clathrin-mediated endocytosis 32. To test whether this mechanism was applicable to our research, we measured the cellular uptake efficiency of M-EXOs by H22 cells with or without incubation of chlorpromazine, which was usually used to inhibit clathrin-mediated endocytosis 33. The flow cytometry analysis revealed that the cellular uptake efficiency of M-EXOs was reduced from 86.15% to 27.07% (Figure S5) when H22 cells were pre-incubated with chlorpromazine. These results demonstrated the main cellular uptake mechanism of M-EXOs was clathrin-mediated endocytosis.

The bio-safety of M-EXOs was evaluated. A Cell Counting Kit-8 (CCK-8) cell viability assay was performed on H22 cells to assess the cytotoxic effects of M-EXOs. In a 640 μg/mL M-EXOs solution, cells maintained as high as 85% viability (Figure 3B). To study the biocompatibility of M-EXOs *in vivo*, hemolytic activity tests were performed to evaluate blood compatibility. No hemolysis was observed (Figures 3C), demonstrating that M-EXOs are biocompatible as a drug delivery vehicle.

### The drug loading and tumor cell inhibition

**Figure 4:**
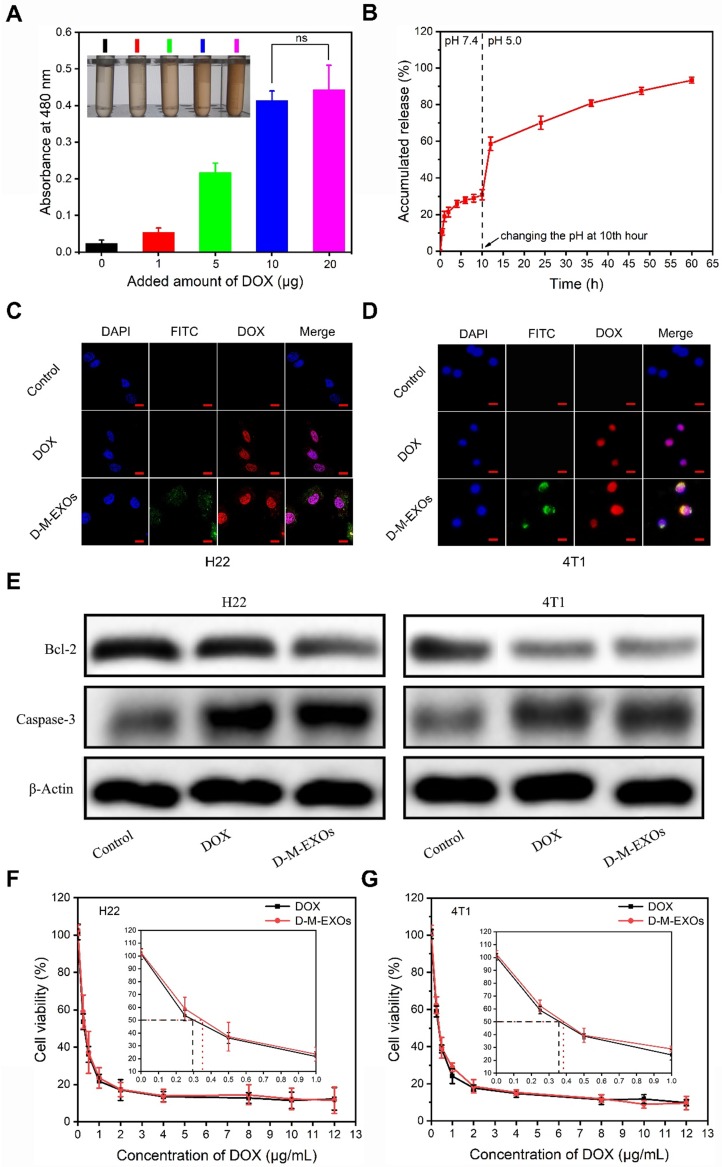
** The drug loading and tumor cell inhibition.** (A) Absorption spectra of D-M-EXOs, with inset showing the image of D-M-EXOs. Significance level is shown as ^ns^p>0.05. (B) Release profiles of DOX from D-M-EXOs. (C) Intracellular distribution of DOX and D-M-EXOs in H22 cells and the bar is 20 μm. (D) Intracellular distribution of DOX and D-M-EXOs in 4T1 cells and the bar is 20 μm. (E) Western blot analysis of Bcl-2 and Caspase-3 protein expression in H22 and 4T1 cells after treatment with DOX and D-M-EXOs. Cell cytoskeleton protein (β-Actin) was used as internal controls. (F) Cell viability of H22 cells exposed to different concentrations of DOX and D-M-EXOs. (G) Cell viability of 4T1 cells exposed to different concentrations of DOX and D-M-EXOs.

The drug loading ability of M-EXOs was tested. Different amounts of DOX were respectively added to M-EXOs solution (100 μg/mL), and the mixed solution was dialyzed to remove un-encapsulated DOX. The DOX-loaded M-EXOs were denoted as D-M-EXOs. The greater the amount of DOX added, the darker the color of the D-M-EXOs solution was (Figure 4A, inserted picture). Furthermore, the ultraviolet-visible absorbance of the D-M-EXOs solution at 480 nm was gradually increased upon increasing the added amount of DOX, and the quantitative analysis indicated a DOX loading capacity of ~10% (Figure 4A).

To evaluate the drug-release behavior of D-M-EXOs, we determined the *in vitro* release profile of DOX from M-EXOs (Figure 4B). The pH of dialysate was changed to more realistically simulate the *in vivo* circulation process of D-M-EXOs. In the first 10 h, D-M-EXOs were placed in PBS dialysate (pH 7.4). The release curve reached a plateau quickly (~77% DOX was retained) indicating M-EXOs could effectively prevent the leakage of DOX in blood circulation. The released DOX (~30%) may be adsorbed on the surface of blood TfR+ exosomes rather than loaded in the phospholipid bilayer. After 10 h, the dialysate was changed to an acetic acid buffer solution (pH 5.0). The releasing rate of DOX speeded up rapidly and ~70% of loaded DOX was released after 24 h. Decreasing the pH resulted in the protonation of DOX and accelerated their release. This result suggested that DOX was released rapidly and massively from M-EXOs after entering an acidic environment, such as late endosomes and lysosomes, of tumor cells. In addition, the result that not all DOX released from M-EXOs in 60 h implied there might be other paths for DOX loading apart from hydrophobic interaction. For example, DOX could be loaded onto nanoparticles by using supramolecular Π-Π stacking 34.

The distribution of D-M-EXOs in tumor cells was examined. Both in H22 cells and 4T1 cells, nearly all DOX was distributed in the nucleus after 24 h of incubation (Figure 4C and 4D). These results implied D-M-EXOs had effective tumor inhibition ability. To prove this conclusion, the expression of Bcl-2 and Caspase-3 was assessed *via* western blot analysis after tumor cells were respectively treated with DOX and D-M-EXOs for 48 h. The DOX and D-M-EXOs groups both showed significantly increased Caspase-3 and an obvious decrease of Bcl-2 compared to the control group (Figure 4E). However, the half-maximal inhibitory concentrations (IC50) of D-M-EXOs on H22 cells and 4T1 cells were ~0.36 μg/mL and ~0.38 μg/mL, slightly higher than that of DOX (~0.29 μg/mL and ~0.35μg/mL) (Figure 4F and 4G). These unsatisfactory results may be due to the incomplete and delayed release of DOX from D-M-EXOs. Although M-EXOs didn't enhance the tumor cell inhibition effect of DOX, they could improve the *in vivo* performance of drugs.

### *In vivo* bio-distribution

**Figure 5:**
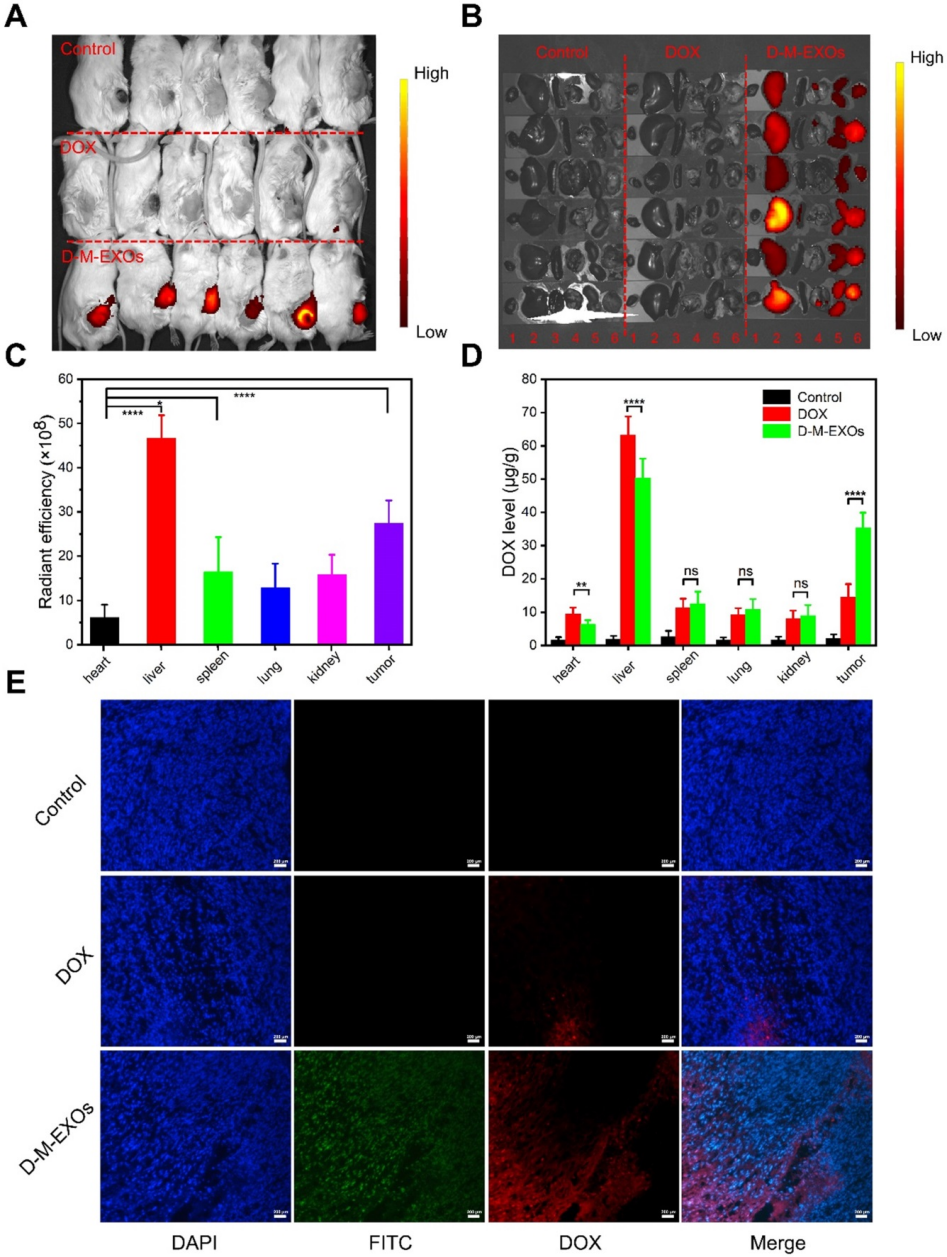
***In vivo* bio-distribution of D-M-EXOs.** (A) Noninvasive NIRF imaging of Cy5.5-labeled D-M-EXOs in Kunming mice after 24 h intravenous injection. (B) Representative *ex vivo* NIRF optical images of tumor and major organs. 1: heart; 2: liver; 3: spleen; 4: lung; 5: kidney; 6: tumor. (C) Radiant efficiency of Cy5.5-labeled D-M-EXOs in tumors and major organs. (D) Levels of DOX in tumors and major organs. (E) Accumulation of FITC-labeled D-M-EXOs in tumor section was evaluated using fluorescence microscopy, and the bar is 200 μm. Significance levels are shown as ^ns^p>0.05,^ *^p<0.05, ^**^p<0.01, and ^****^p<0.001.

The *in vivo* bio-distribution of D-M-EXOs in tumor-bearing mice was tested. As shown in Figure 5A and 5B, the fluorescence signal of D-M-EXOs is observed at tumor sites. Furthermore, the fluorescence intensity in the tumor was higher than that observed in other tissues (heart, spleen, lung, and kidney) except for the liver. The quantitative test result also proved the high accumulation of D-M-EXOs in tumor and liver (Figure 5C). The accumulation of D-M-EXOs in the tumor may be because of the innate passive-targeting ability of blood TfR+ exosomes. In addition, the fluorescence intensity of D-M-EXOs in the liver was much higher, implying their metabolism clearance was mainly through the liver, which was consistent with other researches 29,35. It should be noted that the fluorescence excitation wavelength was selected as 670 nm for the detection of Cy 5.5. However, the fluorescence signal of DOX cannot be excited under this condition. Therefore, no fluorescence signal was observed in the DOX group in the heart and liver, yet this didn't necessarily mean that DOX didn't distribute in the heart and liver.

M-EXOs changed the bio-distribution of DOX. As shown in Figure 5D, when compared with free DOX, D-M-EXOs could enhance the concentration of DOX in the tumor, potentially improving the tumor suppression efficiency. Furthermore, D-M-EXOs reduced the amount of DOX in the heart and liver. This was facilitated to diminish the side effects of DOX. The fluorescence signal of DOX from the D-M-EXOs in the tumor section was much stronger than that of free DOX further indicating that D-M-EXOs improved the tumor-targeting efficiency of DOX (Figure 5E).

### Cardiotoxicity and hepatotoxicity

**Figure 6:**
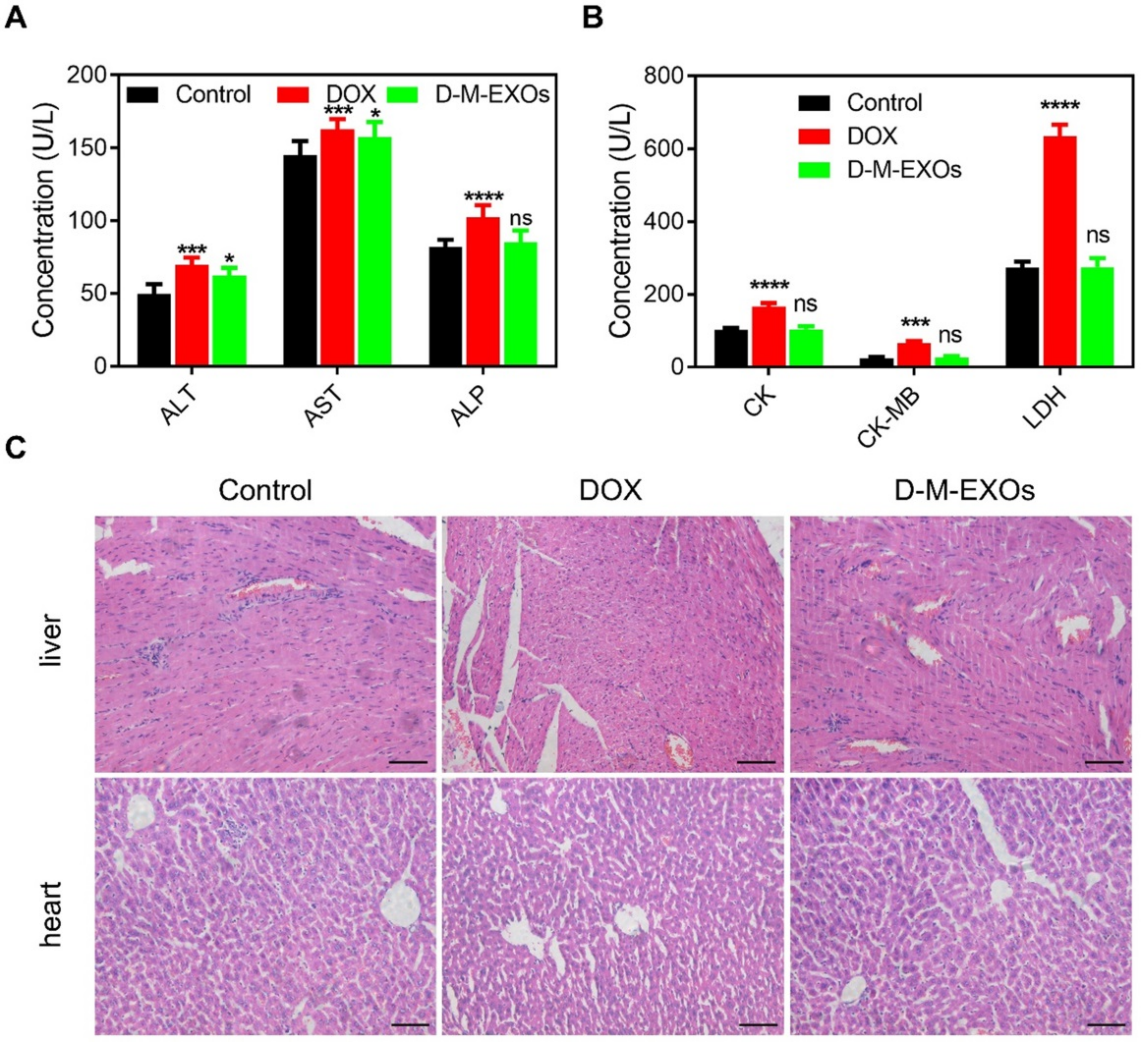
** The cardiotoxicity and hepatotoxicity of D-M-EXOs.** (A) Effects of D-M-EXOs on serum levels of alanine aminotransferase (ALT), aspartate aminotransferase (AST) and alkaline phosphatase (ALP). (B) Effects of D-M-EXOs on serum levels of creatine kinase (CK), creatine kinase-MB (CK) and creatine kinase (LDH). (C) Histological sections of liver and heart stained with H&E, and the bar is 200 μm. Significance levels are shown as ^ns^p>0.05,^ *^p<0.05,^ ***^p<0.005 and ^****^p<0.001.

To verify whether D-M-EXOs indeed reduced the side effects of DOX, their cardiotoxicity and hepatotoxicity were tested. The serum levels of alanine aminotransferase (ALT), aspartate aminotransferase (AST), and alkaline phosphatase (ALP) were respectively measured (Figure 6A). Compared with free DOX, D-M-EXOs didn't obviously induce the expression of ALT, AST, and ALP. In addition, the serum levels of creatine kinase (CK), creatine kinase-MB (CK), and creatine kinase (LDH) in the D-M-EXOs group were much lower than that in the free DOX group (Figure 6B). The serum levels of these factors reflected the function of the liver and heart. The normal expression of these factors indicated that D-M-EXOs showed mild cardiotoxicity and hepatotoxicity. The results of the histological sections of liver and heart stained with H&E (Figure 6C) further proved this conclusion. In addition, the histological sections of other main organs (spleen, lung, and kidney) stained with H&E were also examined (Figure S6). There was no obvious toxicity of DOX in these organs. This may be because the accumulation amount of free DOX in these organs was low and D-M-EXOs can control the release of DOX.

### Tumor suppression by D-M-EXOs

**Figure 7:**
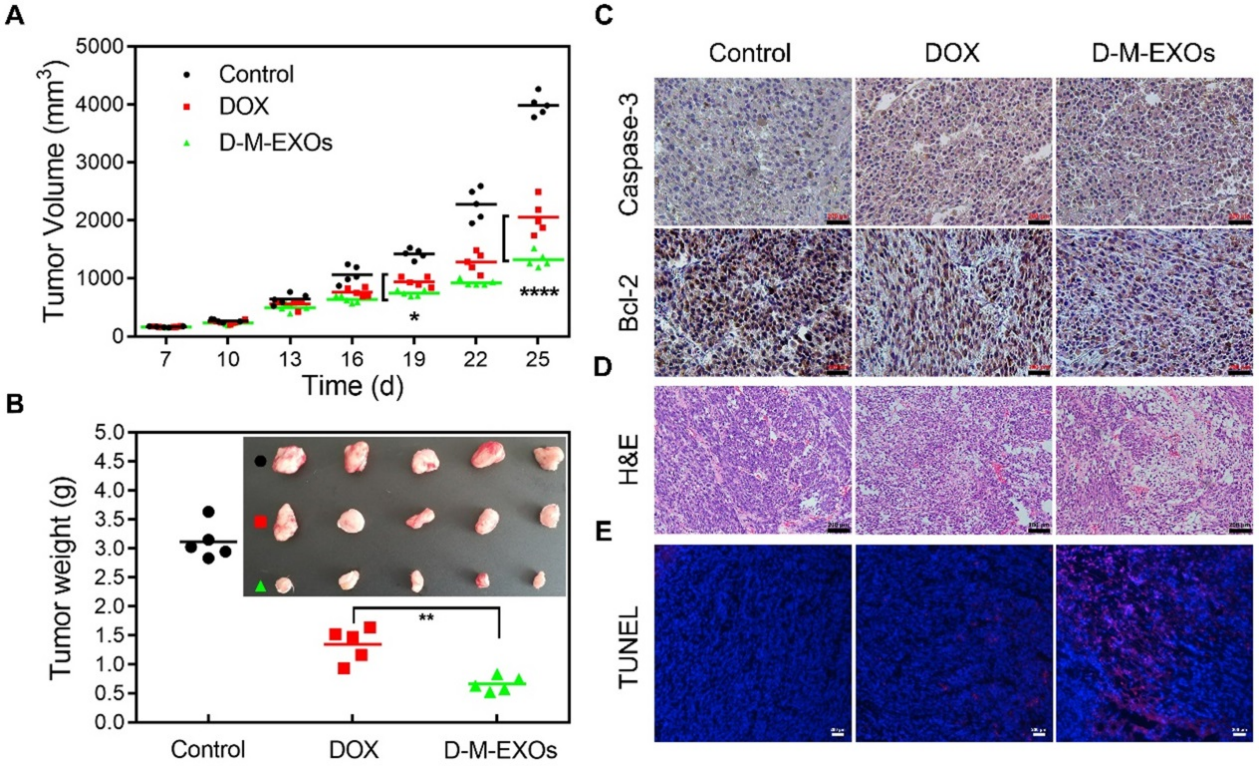
**Efficacy of D-M-EXOs for H22 subcutaneous tumor therapy.** (A) Growth evaluation of H22 subcutaneous tumor in Kunming mice after sample administration; tumor volume was examined every three days for 18 consecutive days. (B) The average mass of the obtained tumor tissues. (C) Immunohistochemistry analyses of the expression of Caspase-3 and Bcl-2 in each group, nuclei are stained blue, and the proteins are stained brown. The bar is 200 μm. (D) H&E staining and (E) TUNEL analysis of the tumor tissues from the mice in each treatment group. In TUNEL staining, normal cell nuclei are stained blue and apoptotic cell nuclei are stained red. Significance levels are shown as ^*^p<0.05, ^**^p<0.01, and ^****^p<0.001.

The tumor inhibition effects of D-M-EXOs on H22-bearing mice were investigated. Mice were injected intravenously with free DOX and D-M-EXOs on days 7, 10, 13, 16, 19, 22, and 25. To examine the kinetics of tumor growth, tumor volume was monitored using a caliper before each injection and calculated as [(length × width^2^)/2]. The tumor volumes of mice treated with PBS increased rapidly within six days (Figure 7A). In contrast, DOX could inhibit the tumor growth to some extent and the tumor volume of mice in the free DOX group was obviously smaller than that in the control group. D-M-EXOs showed a stronger ability to inhibit tumor growth compared with DOX. This result indicated the enhanced bioavailability of DOX as a result of the passive targeting ability of blood TfR+ exosomes. The tumor masses harvested from the mice on day 25 were ~3.12 g, ~1.35 g, and ~0.67 g in mice treated with PBS, DOX, and D-M-EXOs, respectively (Figure 7B). We also examined the expression levels of Caspase-3 and Bcl-2 in tumor tissues harvested from these mice by using immunohistochemistry analyses (Figure 7C). D-M-EXOs could dramatically inhibit tumor growth and up-regulate apoptosis in H22 cells by decreasing Bcl-2 expression and increasing Caspase-3 expression. The fact that D-M-EXOs could effectively trigger the apoptosis of the tumor cells was also confirmed by the direct observation of the slices stained with H&E (Figure 7D) and TUNEL (Figure 7E), respectively. The effective inhibition of tumor growth indicated the feasibility of applying the blood TfR+ exosomes-based drug delivery system.

**Figure 8:**
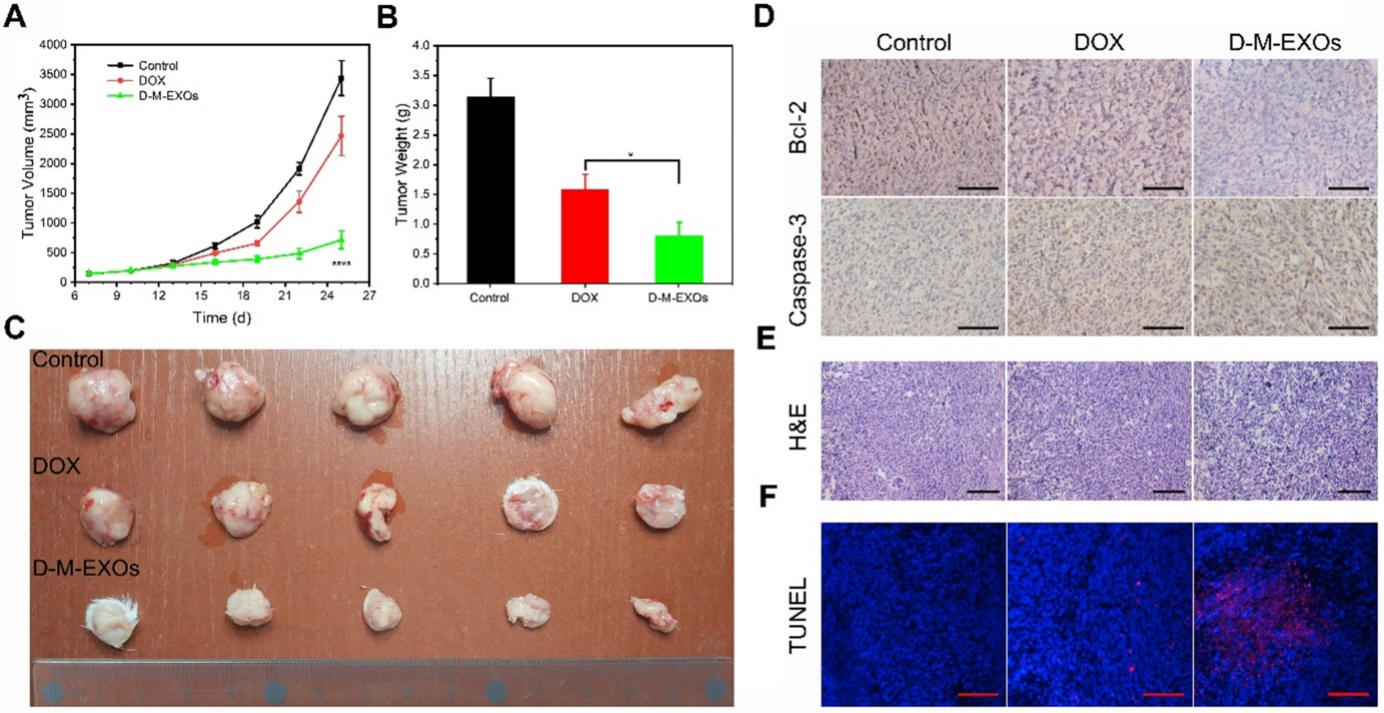
** Efficacy of D-M-EXOs for 4T1 subcutaneous tumor therapy. (**A) Growth evaluation of 4T1 subcutaneous tumor in Kunming mice after sample administration; tumor volume was examined every three days for 18 consecutive days. (B) The average mass of the obtained tumor tissues. (C) 4T1 tumor tissues obtained from euthanized mice 18 days after sample administration. (D) Immunohistochemistry analyses of the expression of Caspase-3 and Bcl-2 in each group, nuclei are stained blue, and the proteins are stained brown. The scale bar is 200 μm. (E) H&E staining and (F) TUNEL analysis of the tumor tissues from the mice in each treatment group. In TUNEL staining, normal cell nuclei are stained blue and apoptotic cell nuclei are stained red. The scale bar is 200 μm. Significance levels are shown as ^*^p<0.05 and ^****^p<0.001.

To further confirm the therapeutic effect, the *in vivo* tumor inhibition effects of D-M-EXOs on 4T1-bearing mice were investigated. The tumor volumes of mice treated with PBS increased rapidly within nine days (Figure 8A). The tumor volume of mice in the free DOX group was obviously smaller than that in the control group indicating the tumor inhibition efficiency of DOX, while D-M-EXOs showed a stronger ability to inhibit tumor growth compared with DOX. The tumor masses harvested from the mice on day 25 were ~3.15 g, ~1.58 g, and ~0.8 g in mice treated with PBS, DOX, and D-M-EXOs, respectively (Figure 8B). Figure 8C was the picture of 4T1 tumor tissues obtained from euthanized mice. The results of immunohistochemistry analyses (Figure 8D), H&E (Figure 8E), and TUNEL (Figure 8F) further proved the effective inhibition of tumor growth by D-M-EXOs.

## Discussion

Here we exploited a kind of pH-responsive method for the separation of blood TfR+ exosomes. In this method, the pH of reaction solutions changed from 7.4 to 5.0 and again to 7.4. In our opinion, the pH change couldn't cause serious damage to exosomes because they went through a similar pH change during their formation process. The inward budding of endosomal membranes led to the accumulation of intraluminal vesicles (ILVs) in large multi-vesicular bodies (MVBs, pH 5.0). Intracellular MVBs can either traffic to lysosomes where they are degraded or to the cell membrane to release ILVs into the extracellular space (pH 7.4). ILVs released into the extracellular space were denoted as “exosomes” 36. Therefore, the separated blood TfR+ exosomes could effectively maintain their structural and functional integrity.

SMNC-EXOs, actually, could be directly applied to drug delivery since the labeled SPMNs had little influence on blood TfR+ exosomes 37. However, the potential cumulative toxicity of SPMNs would limit their long-term clinical application. Blood TfR+ exosomes separated by the pH-responsive SMNC-based method were natural nanocarriers without any modification, avoiding the potential side effects during long-term application. Recently, cells membrane was often used as a coating to shield the surfaces of nanoparticles, such as gold nanoparticles 38, silicon nanoparticles 39, polymeric nanoparticles 40, metal-organic framework nanoparticles 41, and magnetic nanoparticles 42, for enhancing their biocompatibility. Therefore, the high bio-safety of blood TfR+ exosomes was foreseeable because of the similar structure and composition between exosomes membrane and cells membrane. In addition, the successful *in vivo* drug delivery using nanoparticles that were coated with blood cells membrane implied the feasibility of blood TfR+ exosome-based drug delivery. Furthermore, blood cells membrane needed to be extruded during the coating process 43. The membrane structure of blood TfR+ exosomes was potentially more complete than that of blood cells membrane-coated nanoparticles, which might be more conducive to the *in vivo* application of blood TfR+ exosomes. All in all, the high bio-safety of blood TfR+ exosomes is their inherent advantage compared with synthetic nanocarriers.

In some respects, however, the performance of blood TfR+ exosomes is still insufficient when compared to synthetic nanocarriers. As an example, the drug loading capacity of blood TfR+ exosomes was moderate, and some artificial nanoparticles had a better performance. This might because of the difference in the drug loading mode. Current nanoparticles often utilized their large volume and hydrophobic cores to load drugs 44, but it was difficult to access the interior of blood TfR+ exosomes due to their membrane structure. We utilized the hydrophobic interaction between DOX and the lipid bilayer of blood TfR+ exosomes to form D-M-EXOs. Therefore, taking advantage of the internal space of exosomes was a feasible way to further enhance their drug loading capacity.

In addition, the uniformity of blood TfR+ exosomes was poorer than that of synthetic nanoparticles. The size distribution of blood TfR+ exosomes was much broader. More importantly, blood TfR+ exosomes may derive from different cell types. These exosomes may have different properties. For example, chlorpromazine didn't inhibit the cellular uptake of M-EXOs completely. This may be because the cellular uptake pathway of blood TfR+ exosomes derived from varied parent cells was different. Furthermore, even the same exosomes may possess two or more cellular uptake pathways 45.

This work was a continuation of our previous research 37, wherein the focus was on the formation of SMNC-EXOs to simultaneously realize the separation, purification, and tumor targeting of exosomes. In contrast, here, the dissociation of SMNC-EXOs was our purpose, since the lesser modification of exosomes betters their long-term application. How to realize the separation of ligands labeled on SPMNs and receptors expressed on exosomes was the focus. Fortunately, it had been proved that the disaggregation of Tf and TfR was responsive to pH, and blood TfR+ exosomes were widespread in the blood. Therefore, we successfully separated exosomes from blood using a pH-responsive method for the first time. We believed that this study could inspire researchers to exploit new methods for the precise separation of exosomes. For example, other stimulating factors, such as temperature and ion strength, could lead to the disaggregation of ligands and receptors realizing the separation of exosomes.

## Conclusion

In summary, taking advantage of the pH-responsive binding and disaggregation characteristics of transferrin and transferrin receptor, the SMNC-based method can precisely separate blood TfR+ exosomes and realize their mild release from superparamagnetic nanoparticles. The resulting blood TfR+ exosomes show excellent bio-safety and enable the efficient delivery of chemotherapeutics to the tumor. This is the first time that their structural features were taken advantage of to realize the precise and mild separation of blood exosomes. Furthermore, the investigation of the drug delivery potential of blood exosomes facilitates the clinical translation of exosome-based drug delivery systems.

## Abbreviations

DOX: doxorubicin
Tf: transferrin
TfR: transferrin receptor
SPMNs: superparamagnetic nanoparticles
SMNC-EXOs: superparamagnetic nanoparticles-exosome complexes
M-EXOs: blood TfR+ exosomes separated by SMNC-based method
UC-EXOs: blood exosomes separated by ultracentrifugation
D-M-EXOs: doxorubicin-loaded blood TfR+ exosomes

## References

[1] Batrakova EV, Kim MS (2015). Using exosomes, naturally-equipped nanocarriers, for drug delivery. J Control Release.

[2] He C, Zheng S, Luo Y, Wang B (2018). Exosome theranostics: biology and translational medicine. Theranostics.

[3] Alvarez-Erviti L, Seow Y, Yin H, Betts C, Lakhal S, Wood MJ (2011). Delivery of siRNA to the mouse brain by systemic injection of targeted exosomes. Nat Biotechnol.

[4] Tian Y, Li S, Song J, Ji T, Zhu M, Anderson GJ (2014). A doxorubicin delivery platform using engineered natural membrane vesicle exosomes for targeted tumor therapy. Biomaterials.

[5] Wang P, Wang H, Huang Q, Peng C, Yao L, Chen H (2019). Exosomes from M1-Polarized Macrophages Enhance Paclitaxel Antitumor Activity by Activating Macrophages-Mediated Inflammation. Theranostics.

[6] Liu Y, Bai L, Guo K, Jia Y, Zhang K, Liu Q (2019). Focused ultrasound-augmented targeting delivery of nanosonosensitizers from homogenous exosomes for enhanced sonodynamic cancer therapy. Theranostics.

[7] Bellavia D, Raimondo S, Calabrese G, Forte S, Cristaldi M, Patinella A (2017). Interleukin 3-receptor targeted exosomes inhibit *in vitro* and *in vivo* Chronic Myelogenous Leukemia cell growth. Theranostics.

[8] Yim N, Ryu SW, Choi K, Lee KR, Lee S, Choi H (2016). Exosome engineering for efficient intracellular delivery of soluble proteins using optically reversible protein-protein interaction module. Nat Commun.

[9] Zhu L, Kalimuthu S, Gangadaran P, Oh JM, Lee HW, Baek SH (2017). Exosomes derived from natural killer cells exert therapeutic effect in melanoma. Theranostics.

[10] Ohno SI, Takanashi M, Sudo K, Ueda S, Ishikawa A, Matsuyama N (2013). Systemically injected exosomes targeted to EGFR deliver antitumor microRNA to breast cancer cells. Mol Ther.

[11] Kim SM, Yang Y, Oh SJ, Hong Y, Seo M, Jang M (2017). Cancer-derived exosomes as a delivery platform of CRISPR/Cas9 confer cancer cell tropism-dependent targeting. J Control Release.

[12] Lin Y, Wu J, Gu W, Huang Y, Tong Z, Huang L (2018). Exosome-liposome hybrid nanoparticles deliver CRISPR/Cas9 system in MSCs. Adv Sci.

[13] Jang SC, Kim OY, Yoon CM, Choi DS, Roh TY, Park J (2013). Bioinspired exosome-mimetic nanovesicles for targeted delivery of chemotherapeutics to malignant tumors. ACS Nano.

[14] Syn N, Wang L, Sethi G, Thiery JP, Goh BC (2016). Exosome-mediated metastasis: from epithelial-mesenchymal transition to escape from immunosurveillance. Trends Pharmacol Sci.

[15] Kurywchak P, Tavormina J, Kalluri R (2018). The emerging roles of exosomes in the modulation of immune responses in cancer. Genome Med.

[16] Usman WM, Pham TC, Kwok YY, Vu LT, Ma V, Peng B (2018). Efficient RNA drug delivery using red blood cell extracellular vesicles. Nat Commun.

[17] Blanc L, De Gassart A, Géminard C, Bette-Bobillo P, Vidal M (2005). Exosome release by reticulocytes-an integral part of the red blood cell differentiation system. Blood Cells Mol Dis.

[18] Li P, Kaslan M, Lee SH, Yao J, Gao Z (2017). Progress in exosome isolation techniques. Theranostics.

[19] Petersen KE, Manangon E, Hood JL, Wickline SA, Fernandez DP, Johnson WP (2014). A review of exosome separation techniques and characterization of B16-F10 mouse melanoma exosomes with AF4-UV-MALS-DLS-TEM. Anal Bioanal Chem.

[20] Yamada T, Inoshima Y, Matsuda T, Ishiguro N (2012). Comparison of methods for isolating exosomes from bovine milk. J Vet Med Sci.

[21] Liu C, Guo J, Tian F, Yang N, Yan F, Ding Y (2017). Field-free isolation of exosomes from extracellular vesicles by microfluidic viscoelastic flows. ACS Nano.

[22] Tauro BJ, Greening DW, Mathias RA, Ji H, Mathivanan S, Scott AM (2012). Comparison of ultracentrifugation, density gradient separation, and immunoaffinity capture methods for isolating human colon cancer cell line LIM1863-derived exosomes. Methods.

[23] Shao H, Chung J, Lee K, Balaj L, Min C, Carter BS (2015). Chip-based analysis of exosomal mRNA mediating drug resistance in glioblastoma. Nat Commun.

[24] Cheng Y, Zeng Q, Han Q, Xia W (2019). Effect of pH, temperature and freezing-thawing on quantity changes and cellular uptake of exosomes. Protein Cell.

[25] Zhang J, Sun Y, Dong H, Zhang X, Wang W, Chen Z (2016). An electrochemical non-enzymatic immunosensor for ultrasensitive detection of microcystin-LR using carbon nanofibers as the matrix. Sens Actuators B Chem.

[26] Clayton A, Court J, Navabi H, Adams M, Mason MD, Hobot JA (2001). Analysis of antigen presenting cell derived exosomes, based on immuno-magnetic isolation and flow cytometry. J Immunol Methods.

[27] Dautry-Varsat A, Ciechanover A, Lodish HF (1983). pH and the recycling of transferrin during receptor-mediated endocytosis. Proc Natl Acad Sci USA.

[28] Kowal J, Tkach M, Théry C (2014). Biogenesis and secretion of exosomes. Curr Opin Cell Biol.

[29] Qu M, Lin Q, Huang L, Fu Y, Wang L, He S (2018). Dopamine-loaded blood exosomes targeted to brain for better treatment of Parkinson's disease. J Control Release.

[30] Shen Z, Liu T, Li Y, Lau J, Yang Z, Fan W (2018). Fenton-reaction-acceleratable magnetic nanoparticles for ferroptosis therapy of orthotopic brain tumors. ACS Nano.

[31] Blanco E, Shen H, Ferrari M (2015). Principles of nanoparticle design for overcoming biological barriers to drug delivery. Nat Biotechnol.

[32] Tian T, Zhu YL, Zhou YY, Liang GF, Wang YY, Hu FH (2014). Exosome uptake through clathrin-mediated endocytosis and macropinocytosis and mediating miR-21 delivery. J Biol Chem.

[33] Gratton SE, Ropp PA, Pohlhaus PD, Luft JC, Madden VJ, Napier ME (2008). The effect of particle design on cellular internalization pathways. Proc Natl Acad Sci USA.

[34] Wei X, Wang Y, Xiong X, Guo X, Zhang L, Zhang X (2016). Codelivery of a π-π stacked dual anticancer drug combination with nanocarriers for overcoming multidrug resistance and tumor metastasis. Adv Funct Mater.

[35] Wang Ql, Zhuang X, Sriwastva MK, Mu J, Teng Y, Deng Z (2018). Blood exosomes regulate the tissue distribution of grapefruit-derived nanovector via CD36 and IGFR1 pathways. Theranostics.

[36] Théry C, Zitvogel L, Amigorena S (2002). Exosomes: composition, biogenesis and function. Nat Rev Immunol.

[37] Qi H, Liu C, Long L, Ren Y, Zhang S, Chang X (2016). Blood exosomes endowed with magnetic and targeting properties for cancer therapy. ACS Nano.

[38] Gao W, Hu CMJ, Fang RH, Luk BT, Su J, Zhang L (2013). Surface functionalization of gold nanoparticles with red blood cell membranes. Adv Mater.

[39] Parodi A, Quattrocchi N, Van De Ven AL, Chiappini C, Evangelopoulos M, Martinez JO (2013). Synthetic nanoparticles functionalized with biomimetic leukocyte membranes possess cell-like functions. Nat Nanotechnol.

[40] Hu CMJ, Zhang L, Aryal S, Cheung C, Fang RH, Zhang L (2011). Erythrocyte membrane-camouflaged polymeric nanoparticles as a biomimetic delivery platform. Proc Natl Acad Sci USA.

[41] Min H, Wang J, Qi Y, Zhang Y, Han X, Xu Y (2019). Biomimetic Metal-Organic Framework Nanoparticles for Cooperative Combination of Antiangiogenesis and Photodynamic Therapy for Enhanced Efficacy. Adv Mater.

[42] Ren X, Zheng R, Fang X, Wang X, Zhang X, Yang W (2016). Red blood cell membrane camouflaged magnetic nanoclusters for imaging-guided photothermal therapy. Biomaterials.

[43] Rao L, Bu LL, Xu JH, Cai B, Yu GT, Yu X (2015). Red blood cell membrane as a biomimetic nanocoating for prolonged circulation time and reduced accelerated blood clearance. Small.

[44] Xing H, Hwang K, Lu Y (2016). Recent developments of liposomes as nanocarriers for theranostic applications. Theranostics.

[45] Escrevente C, Keller S, Altevogt P, Costa J (2011). Interaction and uptake of exosomes by ovarian cancer cells. BMC Cancer.

