# Blood TfR+ exosomes separated by a pH-responsive method deliver chemotherapeutics for tumor therapy: Erratum

**DOI:** 10.7150/thno.55978

**Published:** 2021-01-01

**Authors:** Lijun Yang, Donglin Han, Qi Zhan, Xueping Li, Peipei Shan, Yunjie Hu, Han Ding, Yu Wang, Lei Zhang, Yuan Zhang, Sheng Xue, Jin Zhao, Xin Hou, Yin Wang, Peifeng Li, Xubo Yuan, Hongzhao Qi

**Affiliations:** 1 Institute for Translational Medicine, Qingdao University, Qingdao 266021, China.; 2 College of Materials Science and Engineering, Qingdao University of Science and Technology, Qingdao 266042, China.; 3 Tianjin Key Laboratory of Composite and Functional Materials, School of Materials Science and Engineering, Tianjin University, Tianjin 300072, China.; 4 School of Clinical Medicine, Weifang Medical University, Weifang 261042, China.

1. In our paper 1, the label of Figure 6C was reversed. The correct version of the figure appears below.

2. In our paper 1, the images of experimental groups in Figure S6 have misused that of the Control group. The correct version of the figure appears below.

The corrections made in this erratum do not affect the original conclusions. The authors apologize for any inconvenience or misunderstanding that this error may have caused.

## Figures and Tables

**Figure 6:**
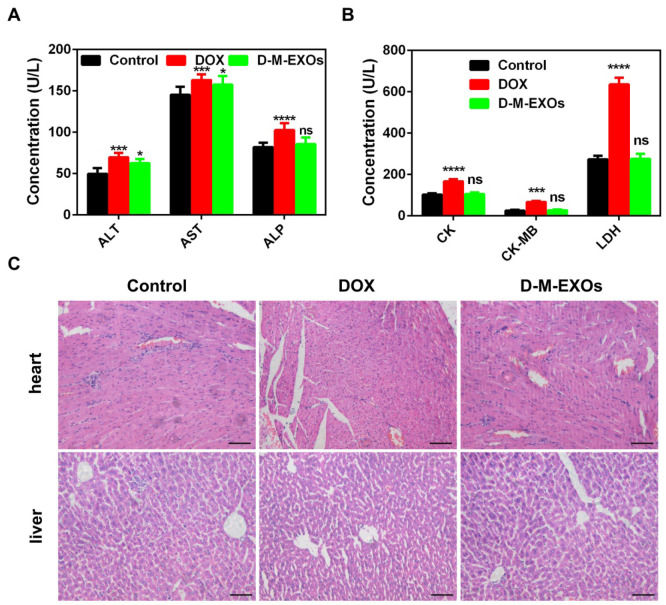
A) Effects of D-M-EXOs on serum levels of alanine aminotransferase (ALT), aspartate aminotransferase (AST) and alkaline phosphatase (ALP); B) effects of D-M-EXOs on serum levels of creatine kinase (CK), creatine kinase-MB (CK) and creatine kinase (LDH); C) histological sections of liver and heart stained with hematoxylin and eosin, the bar is 200 μm. Significance levels are shown as nsp>0.05, *p<0.05, ***p<0.005 and ****p<0.001.

**Figure A:**
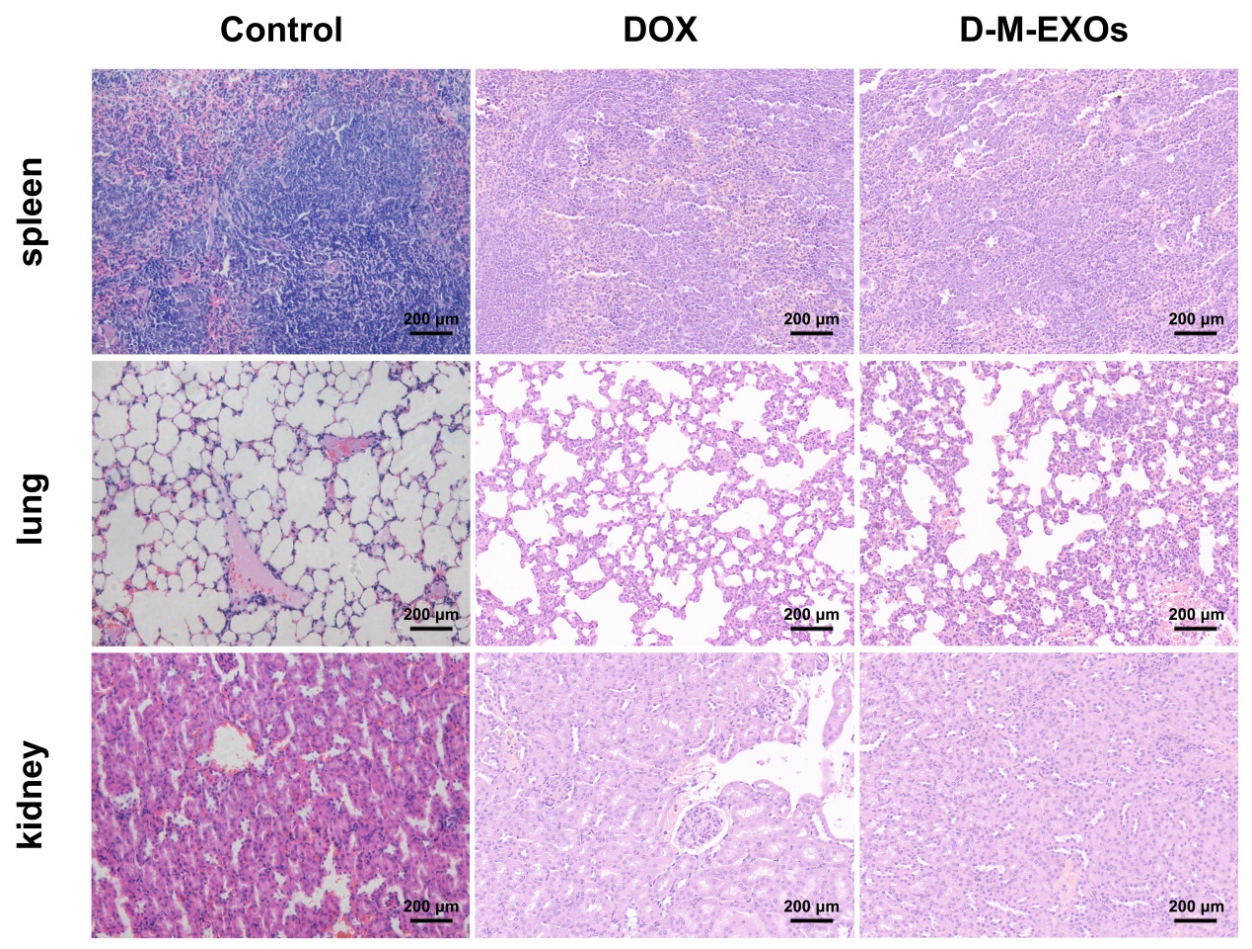
** (Figure S6)** Histological sections of major organs stained with hematoxylin and eosin. The bar is 200 μm.
