# Supplementary material for: Blood TfR+ exosomes separated by a pH-responsive method deliver chemotherapeutics for tumor therapy
Source: Theranostics. 2019 Oct 14;9(25):7680–96. doi: 10.7150/thno.37220 (PMC6831460; doi:10.7150/thno.37220)
Supplement: Supplementary file 1 — Supplementary figures and tables. [file thnov09p7680s1.pdf]

Supporting information

Blood TfR+ exosomes separated by a pH-responsive method deliver chemotherapeutics for tumor therapy

Lijun Yang[2], Donglin Han[3], Qi Zhan[3], Xueping Li[3], Peipei Shan[1], Yunjie Hu[4], Han Ding[1], Yu Wang[1], Lei Zhang[1], Yuan Zhang[1], Sheng Xue[1], Jin Zhao[3], Xin Hou[3], Yin Wang[1], Peifeng Li[1], Xubo Yuan[3,*], Hongzhao Qi[1,*]

1. Institute for Translational Medicine, Qingdao University, Qingdao 266021, China

2. College of Materials Science and Engineering, Qingdao University of Science and Technology, Qingdao 266042, China

3. Tianjin Key Laboratory of Composite and Functional Materials, School of Materials Science and Engineering, Tianjin University, Tianjin 300072, China

4. School of Clinical Medicine, Weifang Medical University, Weifang 261042, China

* Corresponding author: Dr. Hongzhao Qi, Institute for Translational Medicine, Qingdao University, Qingdao 266021, China. E-mail address: Hongzhao Qi (qihongzhao@qdu.edu.cn) or Xubo Yuan (xbyuan@tju.edu.cn)

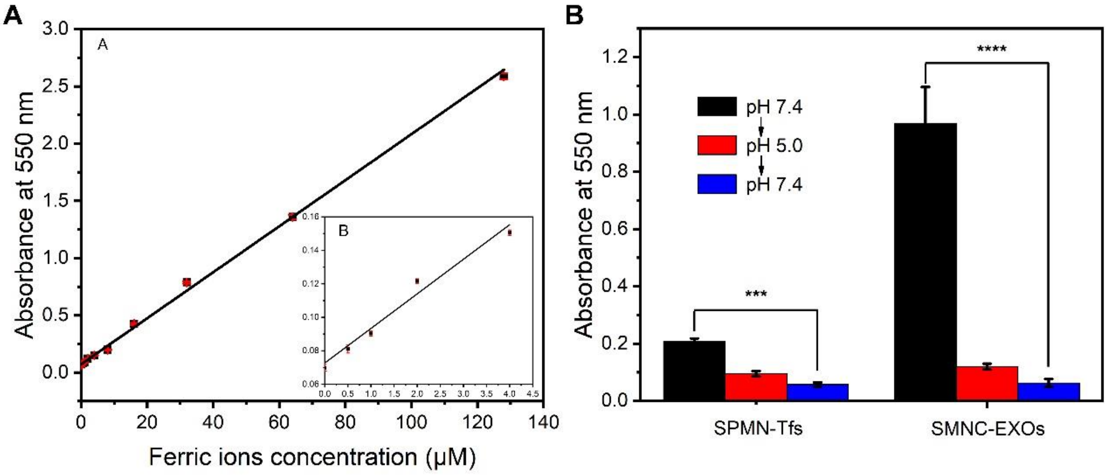

Figure S1. **The ferric ions concentration changes during the preparation process.** (A) The standard curve of the absorbance of the Fe^{2+}-ferrozine complex formed with increasing concentration of the standard $FeCl_3$. (B) The absorbance at 550 nm of Fe^{2+}-ferrozine complex in SPMN-Tfs solution and SMNC-EXOs solution. Significance level is shown as $^{****}p < 0.001$.

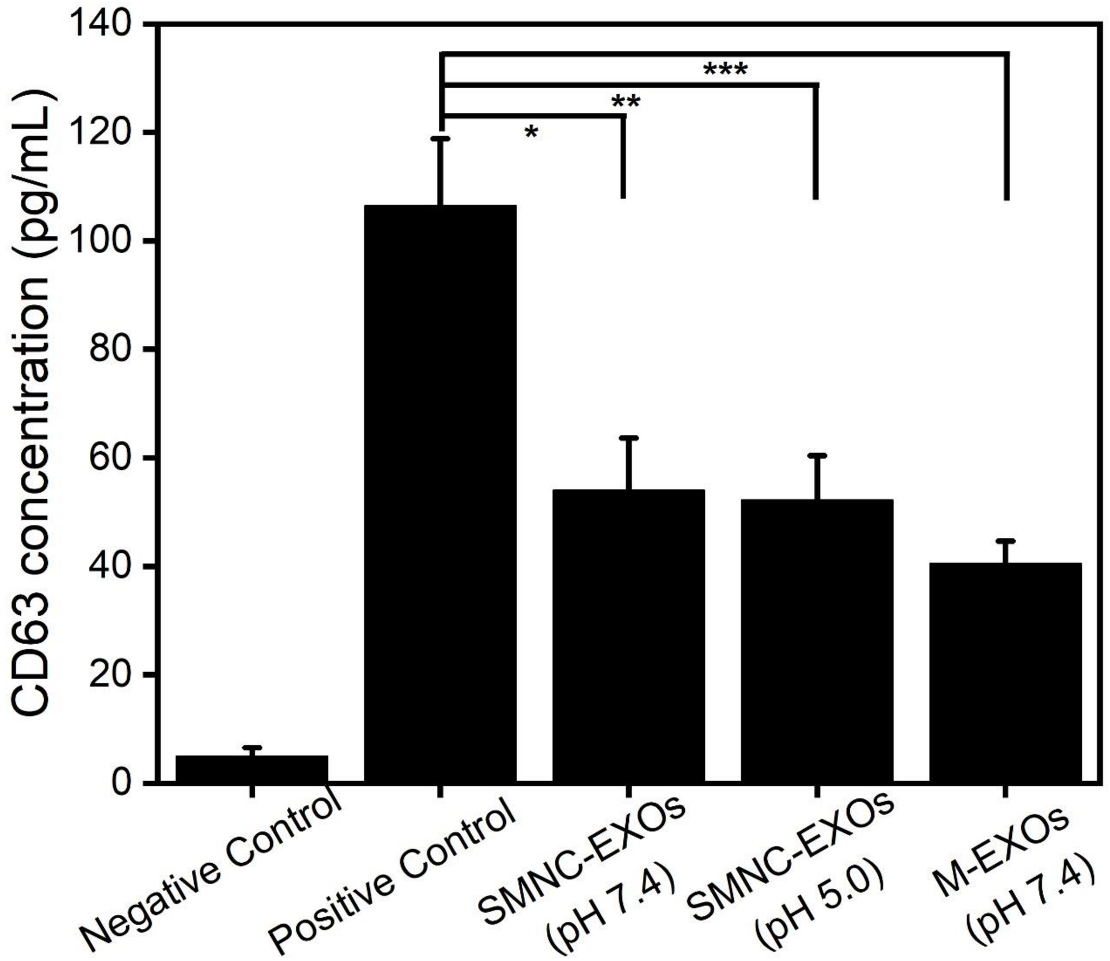

Figure S2. **The change in CD63 concentration during the separation process.** Significance levels are shown as $^{*}p<0.05$, $^{**}p<0.01$, and $^{***}p<0.005$.

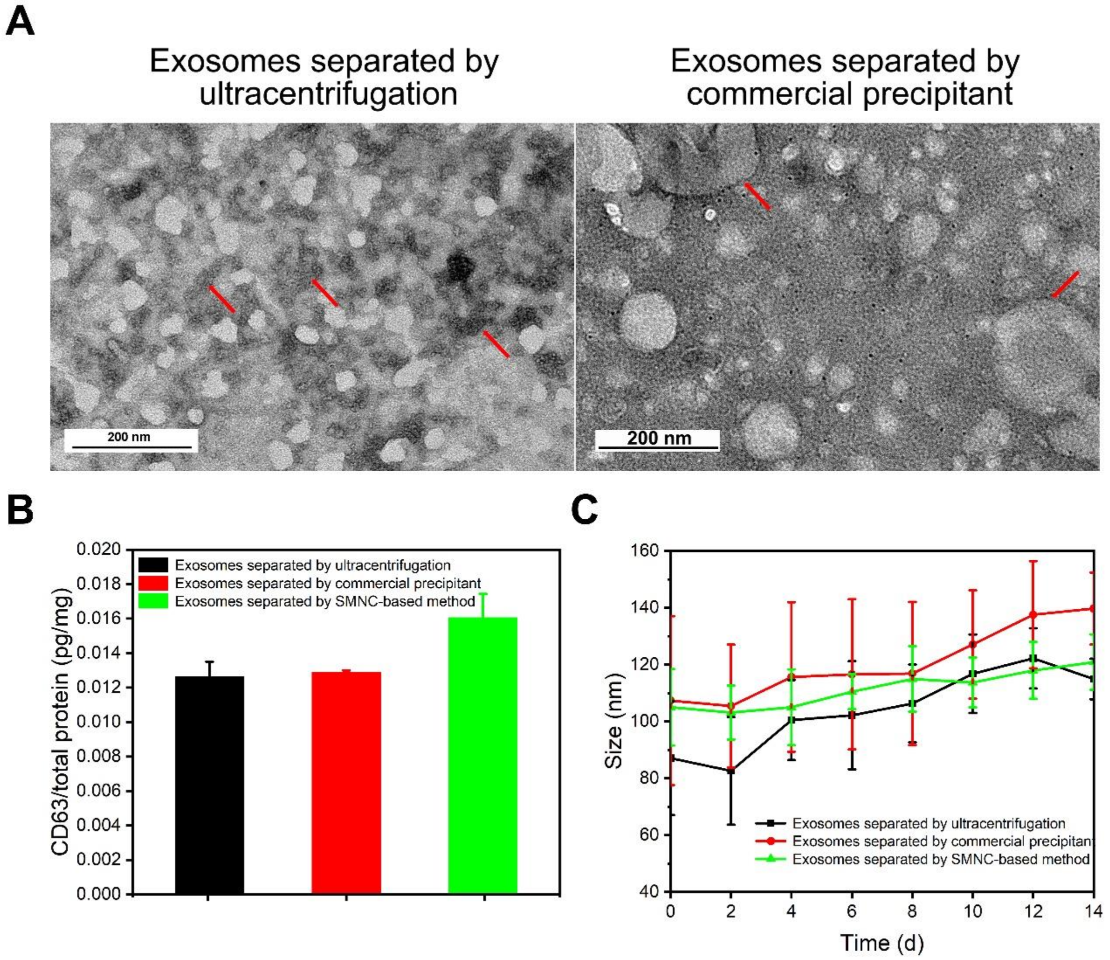

Figure S3. **The comparison of M-EXOs with exosomes that were separated by ultracentrifugation and commercial precipitant.** (A) The representative TEM images of exosomes separated by ultracentrifugation and commercial precipitant. (B) The ratio of the amount of CD63 to that of total proteins in exosomes solution. C) Change in the size of exosomes stored at 4°C in PBS buffer.

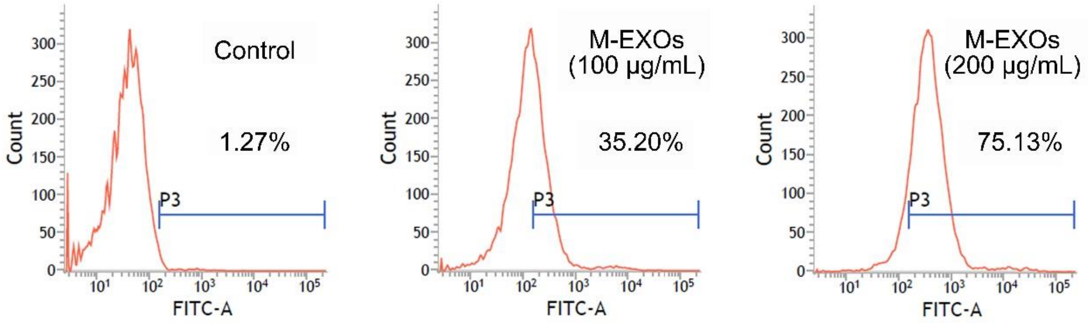

Figure S4. **Flow cytometry analysis of cellular uptake of M-EXOs by H22 cells.**

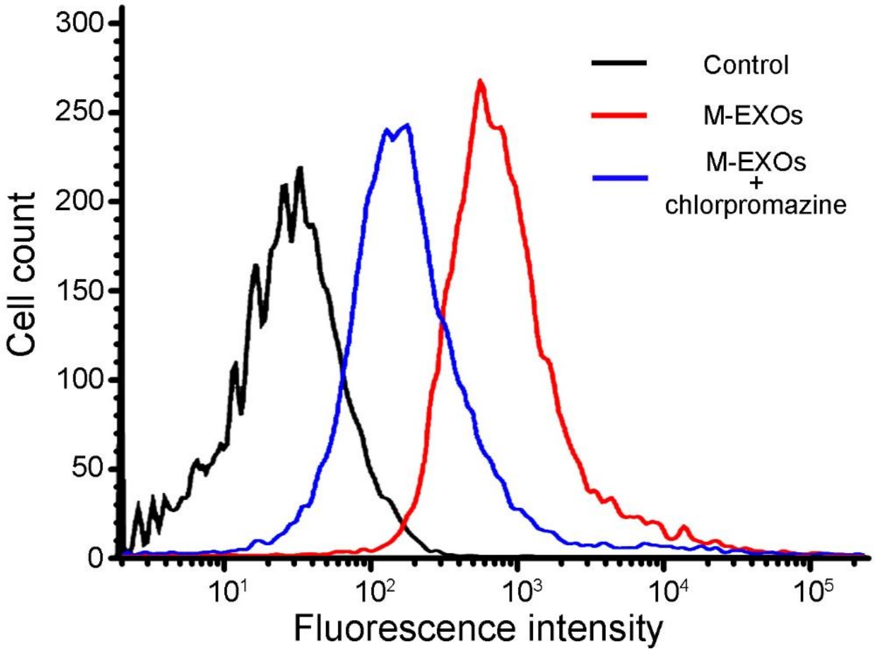

Figure S5. **Flow cytometry analysis of cellular uptake of M-EXOs by H22 cells with or without incubation of chlorpromazine.**

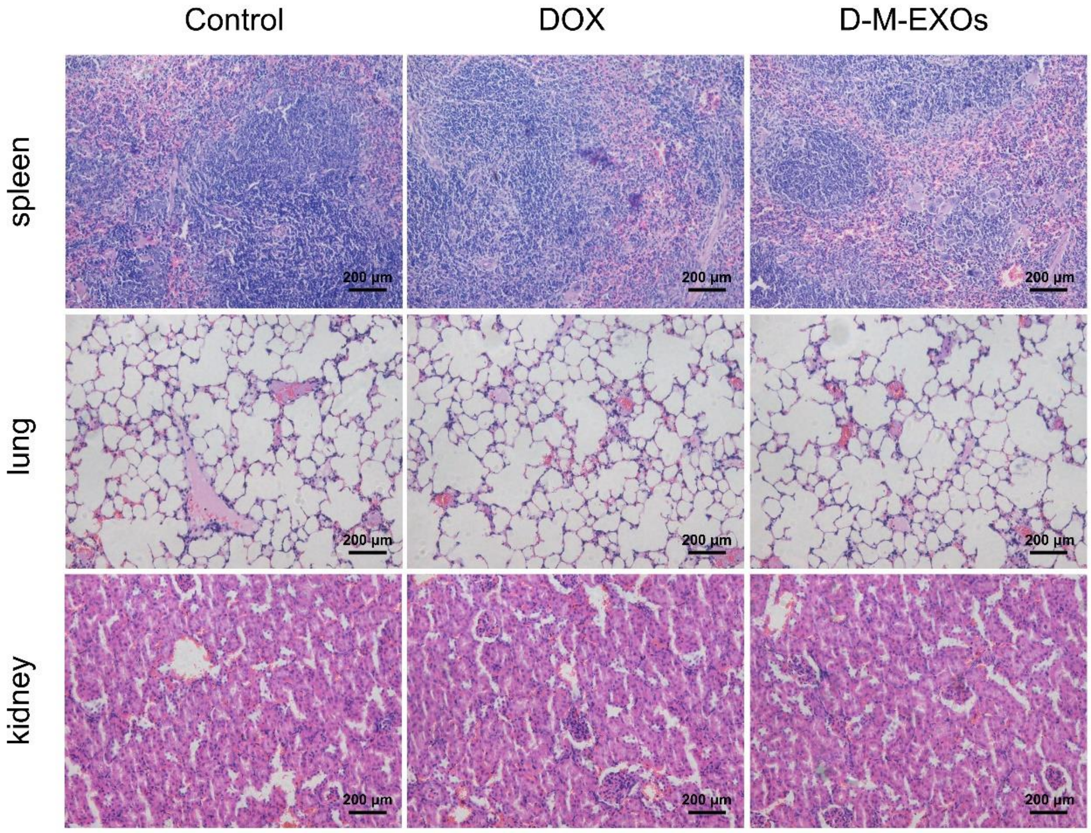

Figure S6. **Histological sections of major organs stained with hematoxylin and eosin (H&E). The bar is 200 μm.**
